# A Refined Model of the Prototypical *Salmonella* SPI-1 T3SS Basal Body Reveals the Molecular Basis for Its Assembly

**DOI:** 10.1371/journal.ppat.1003307

**Published:** 2013-04-25

**Authors:** Julien R. C. Bergeron, Liam J. Worrall, Nikolaos G. Sgourakis, Frank DiMaio, Richard A. Pfuetzner, Heather B. Felise, Marija Vuckovic, Angel C. Yu, Samuel I. Miller, David Baker, Natalie C. J. Strynadka

**Affiliations:** 1 Department of Biochemistry and Molecular Biology, and Centre for Blood Research, University of British Columbia, Vancouver, British Columbia, Canada; 2 Department of Biochemistry, University of Washington, Seattle, Washington, United States of America; 3 Department of Microbiology, University of Washington, Seattle, Washington, United States of America; 4 Department of Genome Sciences, University of Washington, Seattle, Washington, United States of America; 5 Department of Medicine, University of Washington, Seattle, Washington, United States of America; 6 Howard Hughes Medical Institute, University of Washington, Seattle, Washington, United States of America; Osaka University, Japan

## Abstract

The T3SS injectisome is a syringe-shaped macromolecular assembly found in pathogenic Gram-negative bacteria that allows for the direct delivery of virulence effectors into host cells. It is composed of a “basal body”, a lock-nut structure spanning both bacterial membranes, and a “needle” that protrudes away from the bacterial surface. A hollow channel spans throughout the apparatus, permitting the translocation of effector proteins from the bacterial cytosol to the host plasma membrane. The basal body is composed largely of three membrane-embedded proteins that form oligomerized concentric rings. Here, we report the crystal structures of three domains of the prototypical *Salmonella* SPI-1 basal body, and use a new approach incorporating symmetric flexible backbone docking and EM data to produce a model for their oligomeric assembly. The obtained models, validated by biochemical and *in vivo* assays, reveal the molecular details of the interactions driving basal body assembly, and notably demonstrate a conserved oligomerization mechanism.

## Author Summary

Gram-negative bacteria such as *E. coli*, *Salmonella*, *Shigella*, *Pseudomonas aeruginosa*, and *Yersinia pestis* are responsible for a wide range of diseases, from pneumonia to lethal diarrhea and plague. A common trait shared by these bacteria is their capacity to inject toxins directly inside the cells of infected individuals, thanks to a syringe-shaped “nano-machine” called the Type III Secretion System injectisome. These toxins lead to modifications of the host cell, allowing the bacteria to replicate efficiently and/or to evade the immune system, and are necessary to establish an infection. As a consequence, the injectisome is an important potential target for the development of novel therapeutics against bacterial infection. In this study, we focus on the basal body, an essential region of the injectisome that forms the continuous hollow channel across both membranes of the bacteria. We have used an array of biophysical methods to obtain an atomic model of the basal body. This model provides new insights as to how the basal body assembles at the surface of bacteria, and could be used for the design of novel antibiotics.

## Introduction

**Figure 1:**
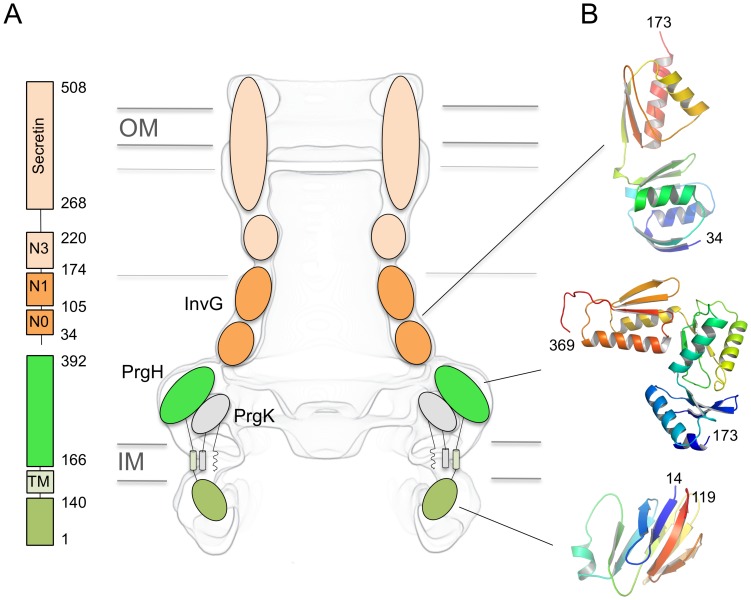
Structures of individual domains from the *Salmonella* SPI-1 basal body. (A) Schematic representation of the proteins composing the basal body. The domain organisation for PrgH (green) and InvG (orange) is shown on the left, and their proposed localization in the EM map of the injectisome is shown on the right. (B) Ribbon representation of the structures of monomeric InvG_22–178_, PrgH_170–392_, and PrgH_11–120_, with their localization in the basal body shown. The boundaries of residues that were observable in the density are indicated.

The bacterial injectisome, or type III secretion system (T3SS), is a specialized syringe-shaped protein-export system utilized by many pathogenic Gram-negative bacteria for the injection of virulence proteins (effectors) into host cells. Bacterial proteins destined for both needle assembly and host cell targeting are translocated via the injectisome in a process known as type III secretion [1]. The injectisome can be divided into three major regions: the inner- (IM) and outer-membrane (OM) spanning basal body with associated export apparatus and ATPase components, an extracellular needle, and a terminating pore inserted into the host cell membrane called the translocon (for review see ref [2], [3]). The major structural scaffold of the basal body is comprised largely of three proteins that arrange into a series of highly oligomerized, concentric rings: two intimately associated proteins localized to the IM - PrgK/EscJ/MxiJ and PrgH/EscD/MxiG, and a third protein belonging to the secretin family of OM proteins, InvG/EscC/MxiD (*Salmonella enterica* serovar Typhimurium SPI-1, Enteropathogenic *Escherichia coli* (EPEC) LEE and *Shigella dysenteriae* nomenclature, respectively) (Figure 1A) [4], [5], [6], [7], [8].

*S.* Typhimurium is an important medical pathogen causing gastroenteritis in infected individuals. Two of the major virulence determining factors are the discrete T3SSs encoded by the *Salmonella* Pathogenicity Islands (SPI) 1 and 2, which are required for bacterial invasion and replication within host cells [9]. The SPI-1 system, belonging to the mxi-spa evolutionary family which also includes the *Shigella dysenteriae* T3SS [10], is considered the prototypical T3SS and has been the focus of structural characterization using a variety of techniques including the first cryo-electron microscopy (EM) 3D reconstruction of a T3SS needle complex [7] revealing its supramolecular assembly. More recently, a cryo-EM analysis of the SPI-1 injectisome at ∼10 Å resolution has provided unprecedented detail of the overall architecture of the basal body [11]. We have previously published the x-ray crystallographically determined structures of the periplasmic domain of the *S.* Typhimurium basal body protein PrgH, in addition to the periplasmic domains of EPEC PrgK homologue EscJ and InvG homologue EscC [6], [12]. These structures defined a common modular domain (ring-building motif; RBM) hypothesized to be involved in ring oligomerization and have been used, along with the available EM data, in all subsequent studies modelling the assembly of these basal body components. The accuracy of these preliminary molecular models [11], [12], [13], however, has been hampered by the use of tentative homology models for some domains, and the lack of structural information entirely for others.

## Results

Here, we make new advances in compiling the precise molecular details of the *Salmonella* SPI-1 injectisome. Specifically, we report the crystal structures of the cytoplasmic domain of the IM ring protein PrgH_11–120_ (and a new crystal form of its periplasmic domain PrgH_170–392_ with improved resolution and detail), as well as the structure of the periplasmic domain of the OM ring InvG. (Figure 1B, Table 1). Collectively these structures provide the most cohesive set of T3SS basal body atomic-resolution structures known from a single species.

**Table 1:** Data collection and refinement statistics for the structures of PrgH_11–120_, PrgH_170–392_ and InvG_22–178_.

		PrgH_11–120_			PrgH_170–392_		InvG_22–178_		
	Hg (peak)	Hg (Inflection)	Hg (remote)	native	native	SeMet (peak)	SeMet (inflection)	SeMet (remote)	native
**Data collection**									
Space group	P21	P21	P21	P21	C2	P212121	P212121	P212121	P212121
Wavelength (Å)	1.0064	1.0087	0.9919	0.979	0.97949	0.97910	0.97931	0.97817	0.97949
**Cell dimensions**									
a, b, c (Å)	33.87, 120.93, 78.11	33.87, 120.93, 78.11	33.87, 120.93, 78.11	33.94, 120.58, 78.28	264.43, 33.38, 48.53	28.43, 56.88, 74.44	28.43, 56.88, 74.45	28.43, 56.87, 74.43	28.27, 56.66, 74.44
α, β, γ (°)	90, 96.7, 90	90, 96.7, 90	90, 96.7, 90	90, 96.47, 90	90, 91.60, 90	90, 90, 90	90, 90, 90	90, 90, 90	90, 90, 90
Resolution (Å)	2.41 (2.55-2.41)	2.41 (2.55-2.41)	2.41 (2.55-2.41)	1.86 (1.96-1.86)	1.85 (1.95-1.85)	2.1 (2.19-2.11)	2.1 (2.19-2.11)	2.1 (2.19-2.11)	1.8 (1.9-1.80)
Rmerge	0.036 (0.105)	0.039 (0.104)	0.043 (0.196)	0.042 (0.227)	0.048 (0.495)	0.106 (0.470)	0.101 (0.441)	0.104 (0.454)	0.066 (0.256)
*I/sI*	16.2 (6.8)	14.6 (6.1)	13.6 (4.4)	10.7 (3.3)	14.9 (2.8)	20.8 (3.9)	17.3 (3.5)	16.9 (3.3)	18.7 (7.3)
Completeness (%)	97 (85.1)	97.1 (88.6)	96.8 (83.7)	96.8 (89.4)	99.5 (99.9)	99.3 (96.0)	98.2 (94.2)	98.7 (96.5)	99.3 (97.4)
Redundancy	2.6 (2.6)	2.6 (2.6)	2.6 (2.6)	2.2 (2.2)	3.7 (3.7)	2.5 (2.3)	1.9 (1.7)	1.9 (1.8)	7.8 (7.5)
**Refinement:**									
Resolution (Å)				1.86	1.85				1.8
No. reflections				50,809	36,630				11,516
*Rwork/Rfree*				0.229/0.256	0.174/0.214				0.190/0.231
No. atoms									
Protein				4,919	3,187				1,147
ions/ligands				1	76				/
Water				58	233				112
B-factors (Å^2^)									
Protein				46.8	40.0				24.1
Water				25.1	45.2				34.1
R.m.s. deviations									
Bond length (Å)				0.017	0.015				0.010
Bond angle (o)				1.83	1.54				1.05

**Figure 2:**
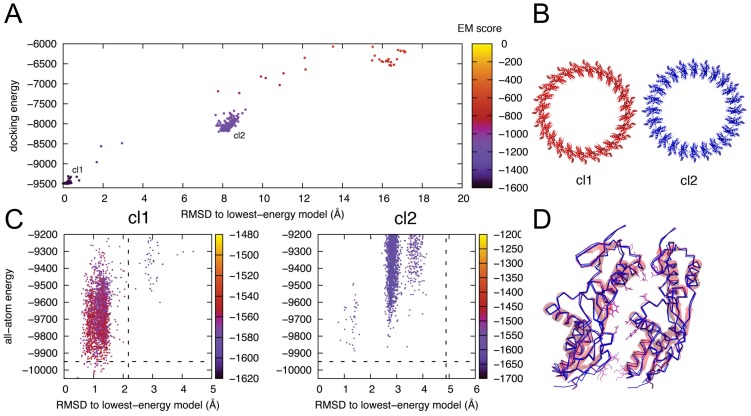
Illustration of the 2-step ring structure modelling approach for the PrgH periplasmic domain. (A) In the first step, fixed-backbone symmetric docking was performed starting from the structure of the monomer. The resulting two clusters of ring arrangements cl1 and cl2 differ in the orientation of the C-terminal domain (B). More sampling was then performed, focusing in the vicinity of the two candidate structures, using perturbation symmetric docking runs (see methods) (C). At this step, the backbone is allowed to move to allow for more efficient energy discrimination. While Cluster 2 quickly diverges from the starting-point seed structure (C-cl2), Cluster 1 runs sink into a lower-energy funnel (C-cl1) (starting structures for the perturbation runs are indicated with vertical dashed lines). The final, low-energy ensemble (indicated below the horizontal dashed line in (B-cl1)) shows highly converged features in terms of the backbone conformation and side-chain packing along the interface (D). Only two adjacent subunits of the 24mer complex are shown for simplicity. The RMSDs are computed for backbone atoms of the entire modelled 24mer complex in (A), while in (C) RMSD values are reported for a single dimeric interface relative to the lowest-energy sampled model. The EM map EMD-1875 is used to restrain both steps of docking calculations, although in the last flexible-backbone stage the weights are reduced to one-half relative to the first, rigid-backbone step.

Using these structures, we have modelled the symmetric ring assemblies by flexible backbone symmetric docking guided by the above EM data (the higher-resolution map EMD-1875 was used unless specified), with the generalized symmetric modelling framework in the program Rosetta [14] (Figures 2 and S1; See Materials and Methods and Appendix S1 for details). This framework makes conformational sampling in symmetric systems tractable by 1) only considering conformations that are consistent with the symmetry of the system and 2) performing a minimal number of energy and derivative evaluations by explicitly simulating only a subset of the interacting monomers and propagating conformational changes to symmetry-related subunits. We implemented a two-step approach to explore symmetric ring conformations consistent with EM data: First, we used an initial global fixed-backbone search starting from a randomized orientation of the monomeric subunits incorporating a score term measuring correlation to the EM data [15], [16] (Figure 2A); this step aims to globally identify ring arrangements that are consistent with the EM data. The candidate fixed-backbone conformations identified in the first step are then explored locally in more detail using symmetric, all-atom refinement with full backbone flexibility [17] (Figure 2B). This step aims at optimizing the symmetric arrangements identified previously by capturing any (small) changes in the backbone conformation. To retain consistency to the EM data, an EM score term with reduced bias is used in the second step. Conformations with the lowest combined score (full atom energy and EM correlation score) from step 2 are then reported as the final models.

For both the cytoplasmic and periplasmic domains of PrgH, we applied a 24-mer oligomerization constraint, according to the established stoichiometry [4], [6], [11]. For the periplasmic domain, we observed that in preliminary modelling runs, residues 361–369 moved during the local perturbation step, and led to a model with a diameter not supported by the EM map, and relatively poor correlation. No visible density was observed for these residues in the crystal form reported previously [12], nor in one of the two molecules in the asymmetric unit of the crystal form reported here, suggesting that these residues are flexible. We therefore performed the docking procedures using residues 173–361 (Figure 2), which led to a model with excellent correlation to the EM map. We note that residues 370–392 were not resolved in any of the crystal forms obtained to date, and therefore these residues were not included in any of the modelling attempts.

The stoichiometry of the T3SS OM secretin has been a matter of debate, with numbers between 12 and 14 proposed [5], [12], [13], [18] and recent studies favouring 12 for the secretins of other systems [19]. Unexpectedly, the recent EM analysis of the SPI-1 basal body suggested a stoichiometry of 15 [11]. We therefore generated ring models with stoichiometries of 12, 14 and 15. To reduce the potential bias of the imposed map averaging, we have carried out the calculations using both the recent high-resolution EM map (EMD-1875) [11] and the previous lower resolution 20× averaged map (EMD-1100) [7]. Based on the docking results, the 12-mer model appears incompatible with the target region of the EM maps [4], having significantly lower interface energies and worse map correlation. Using the lower resolution 20× averaged map, the modelling of the 14- and 15-mer rings results in very similar interfaces with the 14-mer ring having better correlation to the EM map and the 15-mer ring having better all-atom energies. Use of the higher resolution map, however, clearly favours the 15-mer assembly, with the 14-mer configuration having significantly worse correlation to the EM map and Rosetta energy. Assuming a fixed-radius ring, it is expected to have a better-packed interface (and therefore better full-atom energies) for the more compact 15-mer ring than for the 14-mer. The 15-mer shows a similar interface regardless of the map used. Using the high-resolution map, the modelling converged on two opposite modes – a “helix in” conformation and a “helix out” conformation” referring to the orientation of the helix in the N1 domain of InvG (Figure S1B). The “helix in” mode shows excellent agreement with our previous biotinylation [12] and cross-linking [13] experiments, and better fit with the EM map.

**Figure 3:**
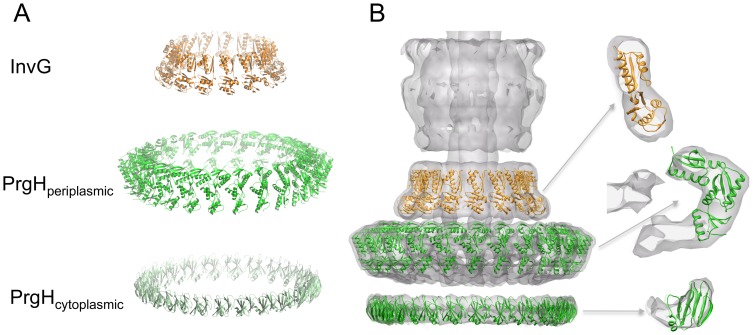
Models of the PrgH and InvG rings. (A) Ribbon representation of the lowest-energy models obtained for PrgH cytoplasmic domain (bottom, dark green), PrgH periplasmic domain (centre, light green) and InvG periplasmic domain (top, orange). (B) Fit of the PrgH (green) and InvG (orange) ring models in the SPI-1 T3SS EM map (EMD-1875) [11]. A close view for the fit of each individual subunit in the EM map density is shown on the right, illustrating the good fit of each subunit within the density.

To demonstrate the importance of using experimentally determined structures for our modelling protocol, we also generated ring models for the cytoplasmic domain of PrgH and the periplasmic domain of InvG using homology models based on the previously available structures MxiG [20] and EscC [12] respectively. For EscC, this strategy led to a model with a similar interface, but with significantly higher energy. In the case of MxiG this led to a model with a different interface, which did not converge during the all-atom refinement step if the PrgH_11–120_ structure was used in a similar conformation (data not shown), thus illustrating how the two-step modelling strategy allows for the discrimination between alternative ring arrangements. For molecular docking applications, the accuracy in the final docking solution is inherently limited by the precision in the backbone structure of the monomeric subunits. In the more general case an accuracy of 2 Å backbone RMSD or better in the coordinates of the monomer is needed to accurately predict the structure of the docked state [21]. Therefore, the availability of the high-resolution crystal structures of the monomeric subunits, rather than homology-based models, combined with the ability to internally rank models based on EM data, enables for high-resolution modelling of the symmetric ring complexes (assuming a minimal degree of change in the conformation of the backbone going from the monomeric to the complex state) (Figure 3, Figure S2).

**Figure 4:**
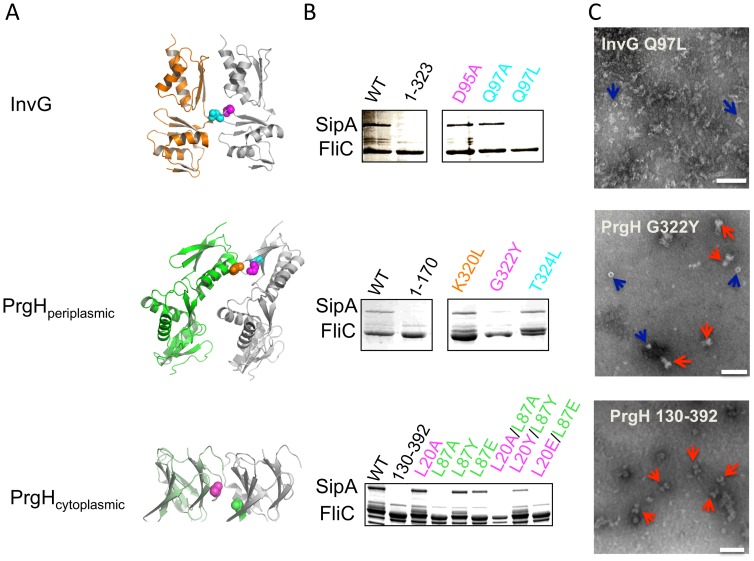
Experimental validation of the ring models. (A) The interface between two subunits for each of the three models is shown. Selected residues involved in contacts are highlighted, which were mutated for secretion assays. (B) Coomassie-stained SDS-page gel of proteins secreted by *S.* Typhimurium strains with a chromosomal deletion of the genes coding for PrgH or InvG, complemented with plasmids containing the corresponding genes, or mutants. The flagellin protein FliC is used as a loading control. The effector protein SipA is secreted when the bacteria express wild-type proteins, but mutation of Gln 97 to leucine in InvG, Gly 322 to tyrosine in PrgH periplasmic domain, or both Leu 20 and Leu 87 to alanine in PrgH cytoplasmic domain show impaired secretion. (C) Electron micrographs of purified T3SS injectisome particles for various InvG or PrgH mutants. Deletion of the cytoplasmic domain of PrgH leads to the formation of injectisome particles that lack the needle (bottom panel, red arrows). Mutation of Gly 322 to tyrosine in the PrgH periplasmic domain (middle panel) leads to the observation of a mix of assembled basal body (red arrow) and individual rings which could consist of either the inner- or outer-membrane components (blue arrows). Finally, mutation of Gln 97 to leucine in InvG severely disrupts the assembly of the T3SS, with only few single rings visible (top panel, red arrows). The white bar indicates 100 nm.

To provide support for our modelled interfaces we have engineered a series of mutant variants and assayed their effect on secretion in *Salmonella* cultures. Mutants were designed using Rosetta to calculate maximal interface disruptiveness and by visual inspection of the models (Figure 4A). For the cytoplasmic domain of PrgH, we observed that mutation of two leucine residues to alanine or an electronegative glutamate (Leu20 and Leu87) abrogates secretion, while a more conservative mutation to tyrosine has no effect (Figure 4B, bottom). For the periplasmic domain of PrgH, we engineered numerous interface and surface mutations (Figure S3). Notably, we identified a loop (residues 319–324) that mediates several side- and main-chain contacts with the adjacent subunit. Side-chain mutations within this loop (K320L, T324L) have no effect on secretion; however, mutation of the conformationally labile Gly322 to a leucine abrogates secretion (Figure 4B, middle), suggesting that the loop main chain conformation/contacts are more critical to interface integrity. Finally, for InvG we observed a loop (residues 95–99) forming a series of side-chain contacts with the adjacent subunit. Mutation of Gln97 within the loop (which forms a hydrogen bond with the adjacent molecule) to leucine – but not alanine - abrogates secretion (Figure 4B, top). Mutation to alanine of the strictly conserved Asp95 within the loop (Figure S4) (which lacks any direct interactions with the neighbouring protomer) has no effect on secretion. In order to support the hypothesis that the observed secretion-deficient phenotypes were due to disruption of the oligomeric interfaces, circular dichroism analysis was used to ensure the introduction of the mutants did not merely deleteriously affect the fold of the individual domains (Figure S5). Further, we purified assembled injectisomes containing mutations in either of the PrgH or InvG domains. Negative stain EM analysis demonstrates that despite being necessary for secretion the PrgH cytoplasmic domain, as observed in a PrgH_130–392_ mutant (lacking the N-terminal cytoplasmic domain), is not required for assembly of the basal body (Figure 4C). However, mutant variant G322Y in the periplasmic domain of PrgH, or Q97L in InvG, disrupts needle complex assembly (Figure 4C).

Clearly, a question that arises from our analysis is the lack of phenotype for several predicted interface mutants, mainly for the PrgH periplasmic domain (Figure S3). One might argue that the complexity of the assembled injectisome, where multiple membrane spanning proteins, filaments and accessory proteins, as well as the membrane environment collectively act to stabilize the basal body rings, making the “all or nothing” action of a single point mutant in the context of the assembled T3SS more difficult. Ideally, the mutant phenotype would be assayed in isolated ring oligomers from individual domains, where they would be expected to be more deleterious. However, this is currently unfeasible due to unsuccessful attempts to reconstitute such T3SS sub-assemblies *in vitro*.

From our analysis, each of the three rings shows an excellent fit to the EM map with correlation coefficients of 0.95, 0.92 and 0.94 for PrgH_cytoplasmic_, PrgH_periplasmic_ and InvG models respectively (Figure 3B, Figure S6 and Video S1). The model of the IM PrgH periplasmic ring is broadly similar to previously reported models from our groups and others [11], [13]. The corresponding region of the EM map is rich in detail and the availability of our previously published crystal structure of this domain facilitated the accurate positioning in the map (Figure S6) and reconstruction of the oligomer [11]. Nonetheless, the average backbone RMSD between the presented model and those previously deposited (PDB ID 2Y9J) is 2.9 Å, corresponding to a small rotation of the monomer subunits (Figure S7). Importantly, our PrgH periplasmic domain model possesses fully refined interfaces (Figure S2B).

For the cytoplasmic domain, the structure of the orthologues from *Chlamydia* (CdsD) (PDB ID: 3GQS), *Shigella* (MxiG) [20], [22] and *Yersina* (YscD) [23], [24] recently confirmed predicted homology to the family of forkhead associated (FHA) domains (Figure S8). Tentative ring models were proposed for MxiG [20], [22]; however, comparison to the model presented here is not possible in the absence of available coordinates for the *Shigella* variant (although from the published figures the models appear to be generally oriented in a similar fashion). FHA domains are frequently involved in interaction with phosphothreonine (pThr)-modified proteins. In *Shigella*, the PrgH orthologue MxiG was reported to interact with phosphorylated peptides from the secretion apparatus protein Spa33 [20] although a separate study failed to corroborate these results [20], [22], and structures of the *Yersinia* orthologue YscD revealed an unconserved phosphothreonine binding motif [23], [24]. Protein sequence alignment of the PrgH orthologues indeed illustrates that the conserved residues involved in phosphopeptide binding in FHA domains are poorly conserved (Figure S9). To validate this, we engineered mutations of the PrgH residues corresponding to the proposed phosphothreonine interacting residues in MxiG - Arg35, Gln42 and Asp65 - to alanine. These mutations have no effect on *in vivo* secretion assays (Figure S10). We therefore conclude that the PrgH cytoplasmic domain is unlikely to interact with pThr-modified proteins. We note however that the FHA phosphopeptide interacting loops in our PrgH structure are accessible on the cytoplasmic face of the ring model, suggesting a potential interface for protein-protein interactions. Indeed, deletion of this domain results in the formation of secretion incompetent immature basal body assemblies lacking needles (Figure 4C) suggesting a role in the correct assembly/coordination of the inner-membrane export apparatus.

For the InvG periplasmic domain, our newly generated ring model is significantly different from existing ones [11], [12], with an average backbone RMSD of over 5.5 Å between our model and the previously deposited one (PDB ID 2Y9K) (Figure S2A and S7)). Importantly, the fully refined interface in our model does not present the atomic clashes present in the previous model. This model was based on homology modelling from the distant EPEC orthologue EscC (22% sequence identity between the periplasmic domains, Figure S4). Superposition of the InvG and EscC crystal structures revealed a similar organization, with the N0 and N1 domains common to the secretin family [19], [25]; however, the relative orientation of these domains is shifted 36 degrees between the two proteins (Figure S11).

Collectively therefore, the basal body model reported here marks a significant advancement from prior models. Of note, the modelling protocol clearly favoured a 15-mer stoichiometry for InvG, in support of recent EM analysis [11]. This result is however in contradiction with the stoichiometry of other secretin proteins [5], [19], [26], [27], [28], and further studies will be required to confirm if this represents a true system-to-system variation.

## Discussion

**Figure 5:**
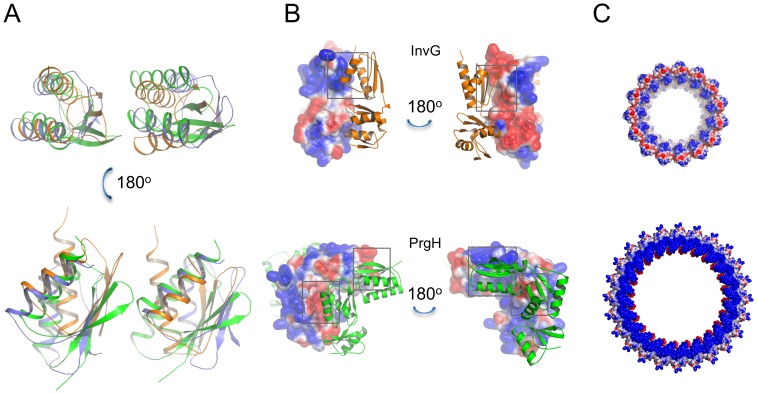
Molecular basis for the SPI-1 basal body assembly. (A) Overlaid view of the interface formed by the RBMs of adjacent molecules for InvG (orange) and PrgH (green for domain 1, blue for domain 2) in the ring models. In all three cases, the orientation of the domains is similar, with the two helices stacking against the three-stranded sheet. (B) Electrostatic surface representation of individual subunits from the PrgH_periplasmic_ (bottom) and InvG (top) ring models. The charged surfaces forming the basis of the interaction between RBMs are indicated in grey boxes. (C) Electrostatic surface representation of the InvG ring model (top) and PrgH periplasmic domain ring model (bottom). The negative charge at the bottom of the InvG ring, as well as the positive charge on the top of the PrgH ring, is shown, suggesting a charge-driven mechanism for the assembly of the *Salmonella* SPI-1 T3SS basal body. The positively charged interior of the InvG N0 ring is also visible.

We have previously observed the presence of a common modular domain, which we termed ring-building motif (RBM), in all the proteins comprising the basal body as well as components of the IM export apparatus [12], [29], [30]. Structural comparison of the intra-subunit interactions of the RBMs in the basal body model supports our previous hypothesis that this domain functions as an oligomerization scaffold, with a conserved interaction mechanism in three RBM mediated interfaces of PrgH and InvG. In all three cases, the conserved interface consists of the N-terminal helix packing against the three-stranded beta sheet of the motif (Figure 5A). Analysis of the electrostatic profile at the RBM interface suggests the driving force for self-association is the complementary charge between the surface formed by the three-stranded beta-sheet and the surface formed by the two helices (Figure 5B). Importantly, similar interactions are present in the oligomeric structure of the EPEC basal body component EscJ [6], suggesting that the RBM plays a common species-independent role in the assembly of the three basal body components. Further, the subsequent observation of an RBM in the T2SS secretin [25], [31] and the intercellular channel complex in sporulating *Bacillus subtilis* [32], [33] suggests this mechanism is likely applicable to related bacterial systems. It should be noted that the N-terminal RBMs of PrgH (defined in the new crystal form of PrgH_170–392_ reported here) and EscJ do not form the conserved oligomerization interface and appear involved in intra-domain interactions or membrane association.

**Figure 6:**
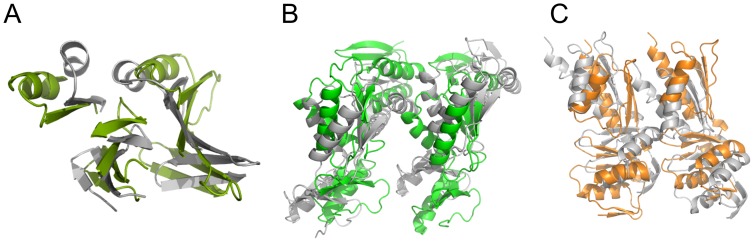
Crystal contacts reflect the oligomerization interface. Overlay of the crystal contacts (grey structures) and the oligomeric interface in the reported ring models, for the cytoplasmic domain of PrgH (A), periplasmic domain of PrgH (B) and periplasmic domain of InvG (C). In all three cases, the crystallographic contacts are formed with an interface that is similar to the interface in the ring model, although the curvature imposed by the oligomeric state differs, leading to some differences in specific side-chain interactions.

All the reported purified domains of the T3SS basal body are monomeric in solution, showing that the membrane-embedded domains and/or the presence of additional proteins are necessary for their oligomerization. Nonetheless, in our earlier structures of the IM protein EscJ from EPEC we observed a 24mer oligomeric packing generated around the 6 fold screw axis of the crystals, the first direct insight into the oligomerization number and interfaces of the ring which has subsequently been supported in re-evaluation of higher resolution EM analysis [11] and has been the basis for all subsequent models of that component and orthologues in the literature. Prompted by this, we investigated if the crystal packing in the structures reported here could be correlated to the interfaces found in our oligomeric models. We observed that the cytoplasmic domain of the second basal body IM ring, PrgH_11–120_, forms a hexamer in the asymmetric unit of the crystal (Figure S12A), with the two interfaces involved in generation of the hexamer being the most sequence conserved surface region (Figure S12B). Comparison of our subsequent Rosetta-EM generated PrgH_11–120_ 24-mer and hexameric crystal packing reveals that the surfaces used for protein-protein contacts is the same in the 6-mer of the crystal form and in the 24-mer of the oligomeric model (Figure 6A). A ∼20° subunit rotation in the hexamer of the crystal structure accommodates the tighter packing but superposition of the interface secondary structural units shows significant conservation with both interfaces having similar buried surface areas (640 Å^2^ vs. 546 Å^2^ for hexamer and 24mer respectively). For the periplasmic PrgH_170–392_ domain, crystallographic symmetry-related molecules in the crystal produce a dimer that exploits the same general interface as the 24-mer biological assembly (Figure 6B). Similarly, a dimer formed by a unit cell translation from the InvG crystal lattice superposes with a dimer from the modelled 15-mer ring (Figure 6C). Collectively, the crystallographic packing interfaces we observe support and mirror the low energy interfaces generated in our modelled basal body. This would be consistent with a model whereby the soluble domains have low-affinity interaction surfaces for oligomerization, which can be captured by the high concentration in the crystallization experiment. *In vivo*, oligomerization is likely dependent on membrane localization and potentially nucleation by additional members of the injectisome.

Finally, analysis of the surface electrostatics of the InvG and PrgH periplasmic ring models reveals an acidic surface present on the face of the InvG ring proximal to PrgH, and a complementary basic surface on the corresponding face of PrgH (Figure 5C). This charge complementarity may provide an initial force of attraction in the assembly of the inner and outer membrane components of the basal body. Furthermore, we observed a positively charged interior collar of the InvG ring corresponding to the N0 domain (Figure 5C), where residues are proposed to be in contact with the inner rod and socket [11], [34]. This surface is formed by a set of basic residues lying on one side of the InvG monomer (Figure S13). In particular, we have previously shown that mutation of Lys67 abrogates secretion by altering substrate switching [13]. From these observations, we can propose a model whereby a large number of weak, charge-based interactions between individual subunits of the basal body lead to a stable complex upon assembly. This is most likely the reason why all the purified soluble domains do not oligomerize *in vitro*, in the absence of the stabilizing trans-membrane domains and lipidic membrane environment. Similar multivalent interactions probably govern the interaction between the basal body and other components of the injectisome, namely the rod and needle. This model is in agreement with the observation that extreme pH conditions lead to the disassembly of the injectisome [7], [11].

While the agreement with the experimental data is compelling, it should be recognized that we have provided models of the ring assemblies, not experimentally determined high resolution structures. In particular, we assume in the initial docking calculations that there are no large scale conformational changes between the monomeric and oligomeric states of individual domains, and that all subunits are identical in the assembled basal body. Furthermore, since the monomers do not spontaneously oligomerize, it is possible that other components, such as the membrane scaffolding, influence the conformation of the oligomeric assembly.

In summary, we report the crystal structures for the cytoplasmic and periplasmic domains of PrgH and InvG, two of the main basal body components of the prototypical *Salmonella* SPI-1 injectisome. We have refined these structures into the EM density using Rosetta symmetric flexible backbone docking calculations, generating ring models for these domains that exhibit a high degree of correlation to the *Salmonella* SPI-1 basal body EM map. The modelling procedure produced converged, low-energy interfaces, which were validated by *in vivo* functional assays. The obtained models provide insights into the discrete interactions occurring during assembly of the T3SS basal body. Analysis of the packing of the previously identified modular domain common to the three basal body components confirms that it represents a common ring-building motif with an electrostatically-driven oligomerization mechanism likely conserved amongst the many clinically important bacteria that rely on a T3SS for their pathogenic effects.

## Materials and Methods

### Cloning, protein expression and purification

The gene coding for PrgH_11–120_ was amplified by PCR from *Salmonella enterica* serovar Typhimurium genomic DNA and cloned into the pET-28(a) plasmid (Novagen) fused to a 6xHis tag at the N-terminus followed by a thrombin cleavage site, using restriction-free PCR [35]. The obtained pET-PrgH_11–120_ was transformed into BL21(DE3) competent cells, and kanamycin-resistant colonies were used to inoculate LB media containing 50 µg/ml of kanamycin, at 37°C to an OD600 of ∼0.5. Expression was induced by IPTG at 0.1 mM, and the protein was expressed at 20°C for 20 hours. Cells were harvested, resuspended in buffer (50 mM TRIS pH 7.4, 300 mM NaCl, 20 mM imidazole), lysed by sonication and debris pelleted at 45,000 g for 50 min. The protein was purified from the supernatant by passing it through Ni-activated chelating sepharose, and the His-tag was subsequently removed by adding thrombin (Roche) at 1∶1000 dilution for 16 hours at 4°C. The protein was further purified by size-exclusion chromatography using a Superdex75 gel filtration column (GE Healthcare). Expression and purification of PrgH_170–392_ was performed as described previously [12].

The gene coding for InvG_22–178_ was amplified by PCR from *S.* Typhimurium genomic DNA and cloned into the pET-28(a) plasmid (Novagen) fused to a 6xHis tag at the N-terminus followed by a thrombin cleavage site, using restriction-free PCR[35]. The obtained pET-InvG_22–178_ was transformed into BL21(DE3) competent cells, and kanamycin-resistant colonies were used to inoculate LB media containing 50 µg/ml of kanamycin, at 37°C to an OD600 of ∼0.5. Expression was induced by IPTG at 1 mM, and the protein was expressed at 37°C for 5 hours. Cells were harvested, resuspended in buffer (50 mM HEPES pH 6.8, 150 mM NaCl), lysed by sonication and debris pelleted at 45,000 g for 50 min. The protein was purified from the supernatant by passing it through Ni-activated chelating sepharose, and the His-tag was subsequently removed by adding thrombin (Roche) at 1∶1000 dilution for 16 hours at 4°C. The protein was further purified by size-exclusion chromatography using a Superdex75 gel filtration column (GE Healthcare).

For SeMet-labelled protein, bacteria were grown in minimal media containing 100 mgs each of added L- Lysine, Phenylalanine, and Tyrosine; 50 mgs of L- Isoleucine, leucine, and valine; and 60 mgs selenomethionine per litre. Purification was performed as for unlabelled protein.

### Crystallization

All initial crystallization trials were performed by sitting-drop vapour diffusion using a Phoenix drop setter (Rigaku). Crystals of PrgH_11–120_ (5–10 mg/ml) were grown at 20°C by sitting-drop vapour diffusion using 15–20% PEG 6000, 0.02 M CaCl_2_, 0.1 M HEPES pH 6.5 as reservoir solution. Crystals of PrgH_170–392_ were obtained at 20°C in a range of conditions using protein samples at 2 mg/ml concentration or less, and all requiring the presence of PEG precipitants. The crystals were all of a new crystal form (compared to those previously-reported by our group [12]), and optimal diffraction was obtained for crystals grown in 100 mM bicine pH 8.5, 20% PEG 6000.

InvG_22–178_ crystallized in several conditions at 20°C, with pH above 7.5 and containing PEG precipitating agents, but formed clusters of needles not amenable for data collection. Single crystals could be obtained by decreasing the protein concentration to approximately 4 mg/ml, and the best diffraction was obtained for crystals grown in 100 mM HEPES pH 8.0, 30% jeffamine M-600. Selenomethionine-labelled protein crystallized similarly.

### Data collection and structure refinement

Crystals were cryo-protected by soaking in the crystallization condition supplemented with 30% glycerol and flash-cooled in liquid nitrogen. Data were collected at beamline 8.1 of the Advanced Light Source (ALS) and beamline CMCF-1 of the Canadian Light Source (CLS) at 100 K. For PrgH_11–120_, a mercury chloride derivative was obtained by soaking in cryo-protectant +2 mM mercury chloride for 10 minutes. A three wavelength MAD experiment was subsequently carried out as well as collection of a high-resolution native dataset in the absence of mercury chloride. For InvG_22–178_ a three wavelength MAD experiment on selenomethionine-derivative crystals was carried out, and a native dataset was collected.

Data were processed and scaled with HKL2000 [36] and MOSFLM [37]/SCALA [38]. For PrgH_170–392_, a molecular replacement solution was found with the program PHASER [39] using the PDB file 3GR0 as a search model, and an initial model was built using ARP/WARP [40]. For PrgH_11–120_ and InvG_22–178_, structure determination and initial model building were carried out with the PHENIX suite [41], which identified 12 Hg atoms for PrgH_11–120_ (FOM 0.51) and 6 Selenium sites for InvG_22–178_ (FOM 0.54). All models were further refined with REFMAC5 [42] and PHENIX, using TLS parameters [43]. Data processing and model refinement statistics are summarized in **Table 1**. Structure quality was assessed with PHENIX and all models have good stereochemistry with PrgH_11–120_, PrgH_170–392_ and InvG_22–178_ having respectively 96.46%, 98.1% and 97.9% of residues in the favoured region of the Ramachandran plot with no outliers. We note the presence of four PEG molecules and four phosphate ions per asymmetric unit in the PrgH_170–392_ crystal structure, for a total of 76 ion/ligand atoms. These molecules were present in the crystallization condition or cryoprotectant buffer, and are therefore not likely to be biologically relevant. The coordinates for PrgH_11–120_, PrgH_170–392_ and InvG_22–178_ have been deposited to the PDB, under the accession numbers 4G2S, 4G08 and 4G1I respectively.

### Ring modelling

Rosetta modelling made use of standard symmetric docking protocols [14], [44] augmented with a term assessing agreement to experimental cryo-EM density [15]. For all symmetric assemblies considered here, symmetric docking calculations were performed in two steps: in a first step, fixed-backbone docking calculations were performed using the monomer conformations from the crystal structures in a randomized orientation. This procedure typically resulted in the identification of a small number of local minima, suggesting potential binding modes for each assembly. In the second, refinement step, we performed a fine-grained local search starting from each binding mode identified in step (1). Here, the rigid body degrees of freedom underwent small random perturbations in a number of Monte-Carlo trajectories. Finally, for each trajectory we performed gradient-based optimization of all backbone, side-chain and rigid body degrees of freedom. The command lines used for each procedure are deposited in Appendix S1. The coordinates for the PrgH_cytoplasmic_, PrgH_periplasmic_ and InvG ring models have been deposited to the PDB, under the accession numbers 3J1W, 3J1X and 3J1V respectively.

### Secretion assay

For complementation assays, mutants were engineered into a plasmid containing the genes coding for PrgH or InvG, described previously [13], [45], using the QuickChange mutagenesis kit (QIAGEN). The obtained plasmids were transformed into electro-competent PrgH- or InvG-deletion strains of *S.* Typhimurium. Secretion assays were performed as described previously [13], [45]. Briefly, 5 ml LB cultures of *S.* Typhimurium strains were grown at 37°C overnight, and cells were then pelleted at 6,000 g for 10 min. Proteins in the supernatant were precipitated by adding 10% TCA and pelleted at 6,000 g for 30 min. Pellets were washed in 0.5 ml acetone, re-suspended in 20 µl gel loading buffer, boiled, and ran in a 10% acrylamide SDS-PAGE gel that was stained with Coomassie Blue.

### Injectisome purification and electron microscopy analysis

Electro-competent strains of *S.* Typhimurium lacking the gene for FliC and either InvG or PrgH [45] were transformed with a plasmid containing the genes coding for PrgH or InvG, described above, or the corresponding mutants. These transformants were used to make electro-competent cells, which were transformed with a plasmid containing the gene coding for the T3SS transcription activator HilA. The obtained transformants were then used for purification of the injectisome, as described previously [45].

For EM analysis, purified injectisome samples were diluted in 10 mM Tris pH 8.0, 500 mM NaCl, 5 mM EDTA and 10 mM LDAO. They were prepared on carbon grids and stained with 0.75% uranyl formate using standard procedures. Images were collected with a H7600 Transmission Electron Microscope (Hitachi Hi-Technologies Canada, Inc.) equipped with a side mount AMT Advantage (1 mega-pixel) CCD camera (Hamamatsu ORCA), and operated at an acceleration voltage of 120 kV.

### Circular dichrosim

Circular dichroism (CD) spectra were recorded with a nitrogen-flushed Jasco J-810 spectro-polarimeter, at 20°C. Proteins were dialyzed against buffer containing 5 mM Tris pH 8 and 50 mM NaCl prior to analysis. 0.1–0.5 mg/ml protein was used with a path length of 0.1 cm. Data were recorded from 260 to 190 nm using a 2 s time constant, 10 nm min^−1^ scan speed and a spectral bandwidth of 1 nm. Spectra were corrected for buffer.

### Structure analysis and representation

The multiple sequence alignments (Figures S4 and S9) were made with ClustalW [46] and the figures generated with ESPript [47]. RMS distances were calculated with PyMol (Schrodinger, LCC). Electrostatic surfaces were calculated with the APBS module [48] in PyMol. Domain angle differences were measured with HingeFind [49]. Conserved residues were mapped on the structures with ConSurf [50]. Map fitting was performed with Chimera [51]. All structure figures were generated with Pymol or Chimera.

## Supporting Information

Figure S1 **Illustration of the 2-step ring structure modelling approach for the PrgH cytoplasmic domain and InvG periplasmic domain.** The results of the fixed-backbone symmetric docking stage and for the perturbation docking calculation are shown for the PrgH cytoplasmic domain (A) and InvG periplasmic domain (B); RMSDs are computed for backbone atoms of the entire modelled 24mer complex for the fixed-backbone docking, while RMSD values are reported for a dimeric interface relative to the lowest-energy sampled model for the perturbation docking calculation. The EM map is used to restrain both steps of docking calculations, although in the last flexible-backbone stage the weights are reduced to one-half relative to the first, rigid-backbone step. (A) Two clusters of arrangements are identified in the fixed-backbone docking stage differing in the orientation of the C-terminus: in cluster 2, the orientation of the monomeric subunit is “flipped” relative to the center of the ring. Cluster 2 is a more collapsed ring that was excluded from further consideration on the basis of its poor fit to the EM map. Cluster 1 was then used as a seed for perturbation docking calculations in which the backbone degrees of freedom are also optimized to allow for more efficient energy discrimination. The final, low-energy ensemble (indicated below the horizontal dashed line) shows highly converged features in terms of the backbone conformation and side-chain packing along the interface. (B) Two clusters of arrangements are identified in the fixed-backbone docking stage differing in the orientation of the C-terminal domain: in cluster 2, the orientation of the monomeric subunit is “flipped” relative to the center of the ring. Cluster 2 was excluded from further consideration based on biochemical data (see the text). In the flexible-backbone step starting from cluster 1 (vertical dashed line), we obtain a highly converged low-energy interface (indicated below the horizontal dashed line).
(TIF)

Figure S2 **The well-packed interface of the PrgH and InvG ring models.** The ring models of InvG periplasmic domain (A, orange), PrgH periplasmic domain (B, green) and PrgH cytoplasmic domain (C) are shown on the left, overlaid on the models reported previously (in blue and purple, for (A) and (B) respectively). A close-up of the interface between molecules is shown on the right, with the interface side-chains apparent. For all three models, the interfaces are well packed, with a relevant network of interactions.
(TIF)

Figure S3 **Mutants generated for the PrgH periplasmic domain.** The location of all the mutated residues that were tested for secretion assay (K218A, K218L, E227L, D251A, D251L, I234A, I234L, Y239A, R231Y, I234R, Y238F, R262A, R262L, Q263A, Q263L, D295A, D295L, V297R, K308L, Q309Y, K320L, G322Y, T324H, K218A/D251A, K218A/Y239A, K218L/Y239A, K218L/Y239F, Y239A/E252A) is indicated on a PrgH molecule shown in cartoon representation, with the adjacent molecules in the ring model shown in surface representation. Only the G322Y mutant abrogated secretion (see Figure 4B)
(TIF)

Figure S4 **Multiple alignment of the sequences from the secretins of several T3SSs.** InvG_SPI1: *S.* Typhimurium SPI-1 secretin InvG; EivG_ETT2: Enterohaemorrhagic *E. coli* ETT2 secretin EivG; MxiD_Shi: *Shigella* secretin MxiD; YscC_Yer: *Yersinia pestis* secretin YscC; PscC_Pse: *Pseudomonas aeruginosa* secretin PscC; EscC_LEE: Enteropathogenic *E. coli* LEE secretin EscC; SsaC_SPI2: *S.* Typhimurium SPI-2 secretin SsaC. Strictly conserved residues are in a red box, similar residues are in red characters. The secondary structures for the periplasmic domains of InvG and EscC are in blue (top) and green (bottom) respectively.
(TIF)

Figure S5 **Circular dichroism analysis of InvG_22–178_, PrgH_170–392_ and PrgH_11–120_.** Circular dichroism (CD) spectra of the crystallized domains of InvG and PrgH demonstrate the designed interface destabilizing mutants PrgH_11–120_ L87A (A), PrgH_170–392_ G322L (B) and InvG_22–178_ Q97L (C) have minimal effect on the overall secondary structure. CD is reported in mdeg and overlaid spectra have been corrected for protein concentration.
(TIF)

Figure S6 **Fit of the ring models into the EM map.** Close view of a monomer from the InvG (top), PrgH_periplasmic_ (middle) and PrgH_cytoplasmic_ (bottom) ring models, into the EM map density (EMD-1875). Side views (left) and top views (right) are shown, and a lower contour level (0.075 for InvG, 0.08 for PegH_periplasmic_ and 0.045 for PrgH_cytoplasmic_) compared to figure 3B is used for the EM density, for closer illustration of the fit quality.
(TIF)

Figure S7 **Comparison with previous models.** The RMSD between monomers in our PrgH_periplasmic_ and InvG models (green and orange respectively) and the models reported by Schraidt and Marlovits (grey) were calculated using PyMol. The difference in atom position is shown in yellow bars, illustrating the rotation in the PrgH model and the dramatic difference in the InvG model.
(TIF)

Figure S8 **Comparison of the C-terminal domain or PrgH with its orthologues.** Ribbon representation of PrgH_11–120_ (green), overlaid with the structures of its orthologues MxiG (pdb:2XXS, orange) from *Shigella*, and CT664/CdsD (pdb: 3GQS, blue) from *Chlamydia*, viewed from the side of the beta-sandwich (left) and from the top (right). The common FHA fold is illustrated, with the variable single helix clearly apparent.
(TIF)

Figure S9 **Multiple alignment of the sequences from PrgH orthologues in several T3SSs.** PrgH_SPI1: *S.* Typhimurium SPI-1 protein PrgH; EprH_ETT2: Enterohaemorrhagic *E. coli* ETT2 protein EprH; MxiG_Shi: *Shigella* protein MxiG; YscD_Yer: *Yersinia pestis* protein YscD; PscD_Pse: *Pseudomonas aeruginosa* protein PscD; EscD_LEE: Enteropathogenic *E.coli* LEE protein EscD; SsaD_SPI2: *S.* Typhimurium SPI-2 protein SsaD. Strictly conserved residues are in a red box, similar residues are in red characters. The secondary structure of the cytoplasmic and periplasmic domains of PrgH is shown at the top (blue), and of the periplasmic domain of MxiG at the bottom (green). Red stars indicate proposed phThr-binding residues.
(TIF)

Figure S10 **The putative pThr-binding residues of PrgH are not necessary for secretion.** Coomassie-stained SDS-page gel of proteins secreted by *S.* Typhimurium strains with a chromosomal deletion of *PrgH*, complemented with a plasmid containing the *PrgH* gene or mutants. The flagellin protein FliC is used as a loading control. Secretion is not affected by the R35A, Q42A or D65A mutations, demonstrating that these residues are not essential for function.
(TIF)

Figure S11 **Structural differences between InvG and EscC.** Ribbon representation of the structure of InvG_22–178_ (orange), overlaid onto the structure of EscC_21–174_ (cyan). The overlay was based on the N1 domains, illustrating the shift in relative orientation for the N0 domain, viewed from the side (left) or front (right).
(TIF)

Figure S12 **PrgH_11–120_ crystallizes as a hexamer.** Ribbon representation of the PrgH_11–120_ crystal asymmetric unit. (A) The six molecules form a ring, with the helix located in the lumen. (B) Residue conservation is indicated on the surface representation, with the most conserved residues shown in red and the least conserved residues in blue. Conserved residues are primarily located at the interface between molecules, suggesting that this interface is biologically relevant.
(TIF)

Figure S13 **Molecular basis for the charge repartition in the InvG ring model.** Ribbon representation of the InvG_22–178_ monomer, with the residues forming the basic lumen of the InvG ring shown in blue, and those forming the acidic patch at the bottom of the InvG ring shown in red.
(TIF)

Appendix S1 **Detailed protocol for the two-step modelling procedure.** (A) Schematic representation of the procedure employed. (B) Command lines used in Rosetta for the modelling procedure.
(DOC)

Video S1 Ring models of the PrgH cytoplasmic domain (dark green), PrgH periplasmic domain (green) and InvG periplasmic domain (orange) docked in the EM map of the injectisome (EMD-1875, grey).
(MOV)
