# Supplementary figures and images for: A Refined Model of the Prototypical Salmonella SPI-1 T3SS Basal Body Reveals the Molecular Basis for Its Assembly
Source: PLoS Pathog. 2013 Apr 25;9(4):e1003307. doi: 10.1371/journal.ppat.1003307 (PMC3635987; doi:10.1371/journal.ppat.1003307)

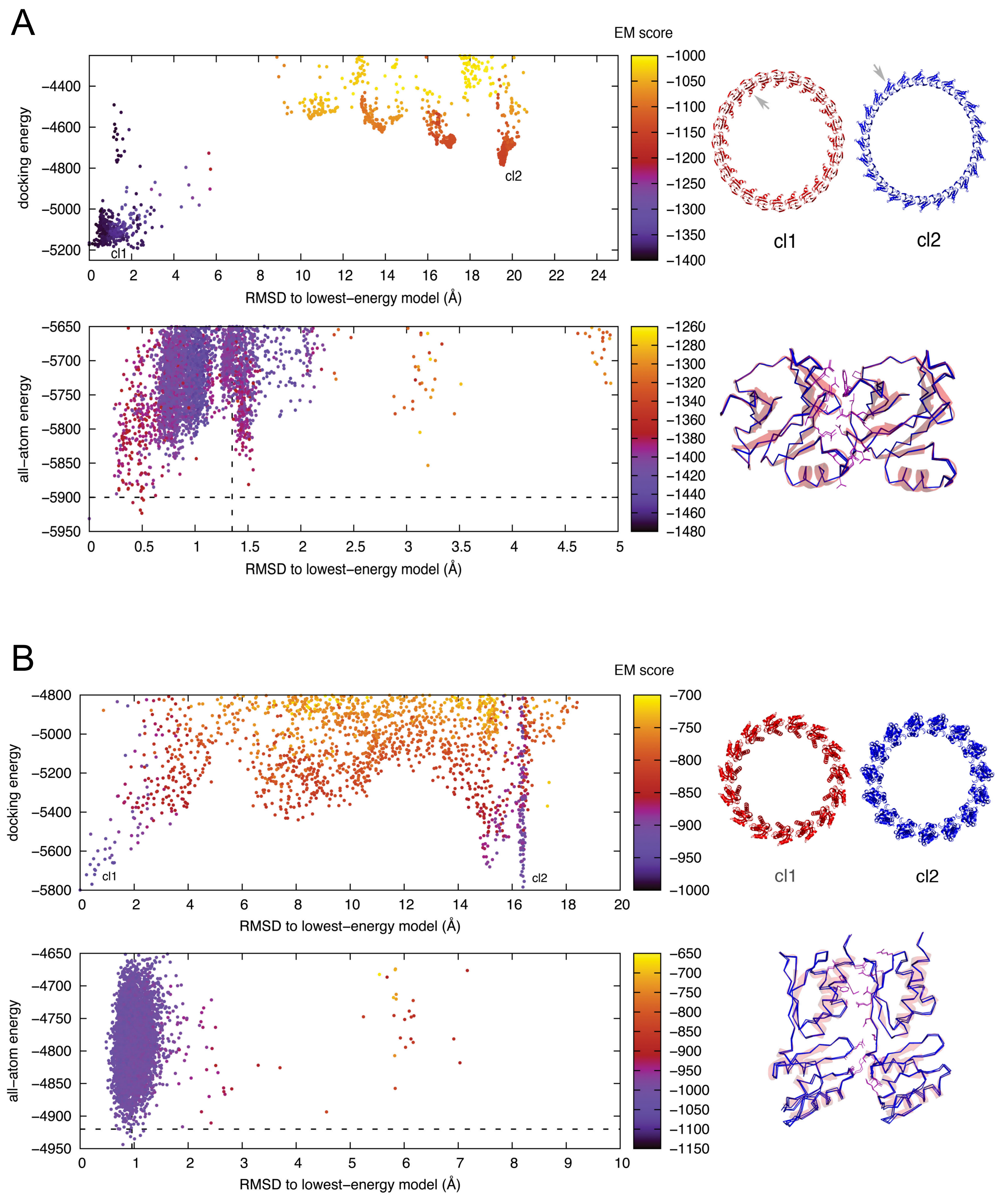

Supplement: Figure S1 — Illustration of the 2-step ring structure modelling approach for the PrgH cytoplasmic domain and InvG periplasmic domain. The results of the fixed-backbone symmetric docking stage and for the perturbation docking calculation are shown for the PrgH cytoplasmic domain (A) and InvG periplasmic domain (B); RMSDs are computed for backbone atoms of the entire modelled 24mer complex for the fixed-backbone docking, while RMSD values are reported for a dimeric interface relative to the lowest-energy sampled model for the perturbation docking calculation. The EM map is used to restrain both steps of docking calculations, although in the last flexible-backbone stage the weights are reduced to one-half relative to the first, rigid-backbone step. (A) Two clusters of arrangements are identified in the fixed-backbone docking stage differing in the orientation of the C-terminus: in cluster 2, the orientation of the monomeric subunit is “flipped” relative to the center of the ring. Cluster 2 is a more collapsed ring that was excluded from further consideration on the basis of its poor fit to the EM map. Cluster 1 was then used as a seed for perturbation docking calculations in which the backbone degrees of freedom are also optimized to allow for more efficient energy discrimination. The final, low-energy ensemble (indicated below the horizontal dashed line) shows highly converged features in terms of the backbone conformation and side-chain packing along the interface. (B) Two clusters of arrangements are identified in the fixed-backbone docking stage differing in the orientation of the C-terminal domain: in cluster 2, the orientation of the monomeric subunit is “flipped” relative to the center of the ring. Cluster 2 was excluded from further consideration based on biochemical data (see the text). In the flexible-backbone step starting from cluster 1 (vertical dashed line), we obtain a highly converged low-energy interface (indicated below the horizontal dashed line). (TIF) [file ppat.1003307.s001.tif]

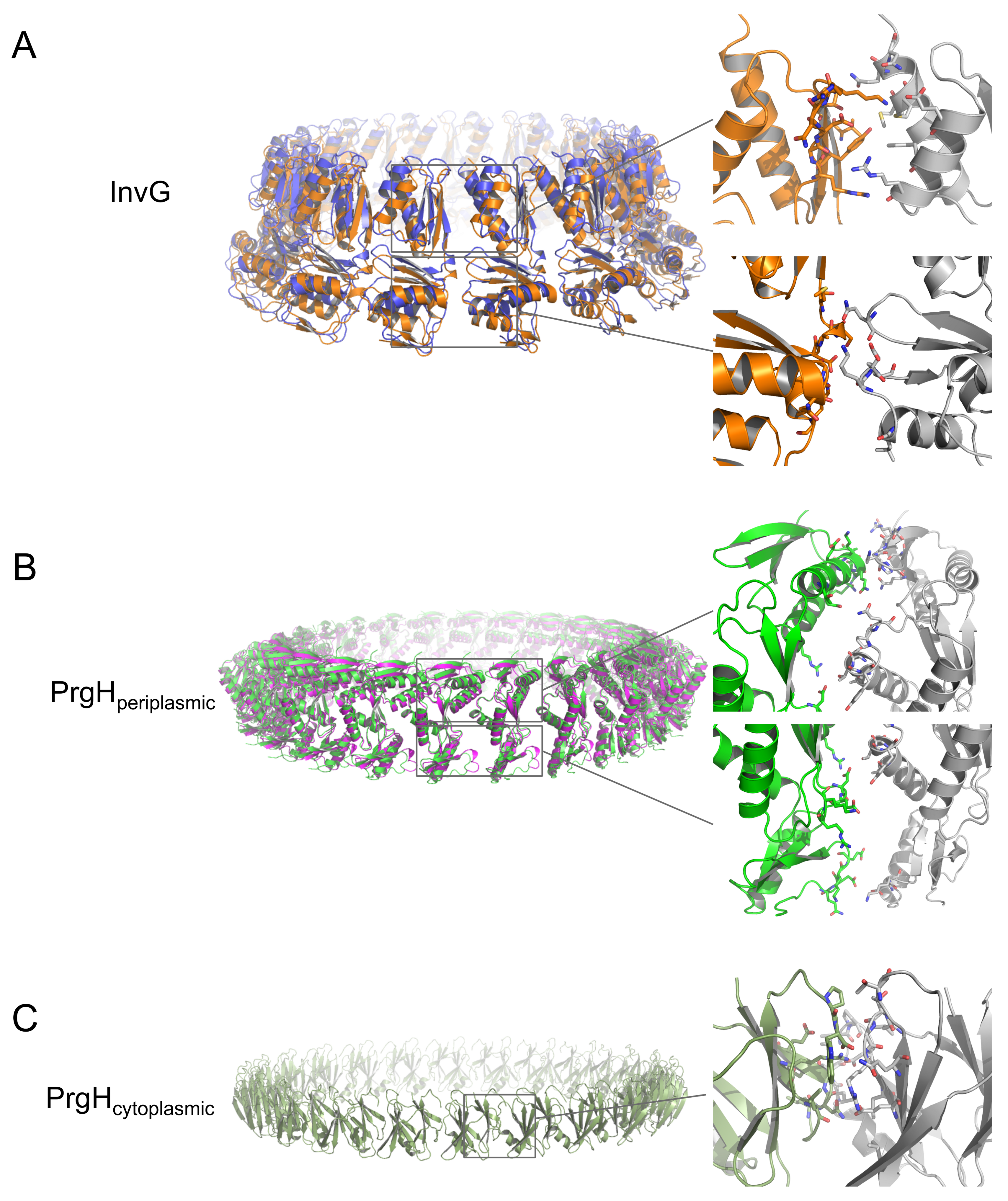

Supplement: Figure S2 — The well-packed interface of the PrgH and InvG ring models. The ring models of InvG periplasmic domain (A, orange), PrgH periplasmic domain (B, green) and PrgH cytoplasmic domain (C) are shown on the left, overlaid on the models reported previously (in blue and purple, for (A) and (B) respectively). A close-up of the interface between molecules is shown on the right, with the interface side-chains apparent. For all three models, the interfaces are well packed, with a relevant network of interactions. (TIF) [file ppat.1003307.s002.tif]

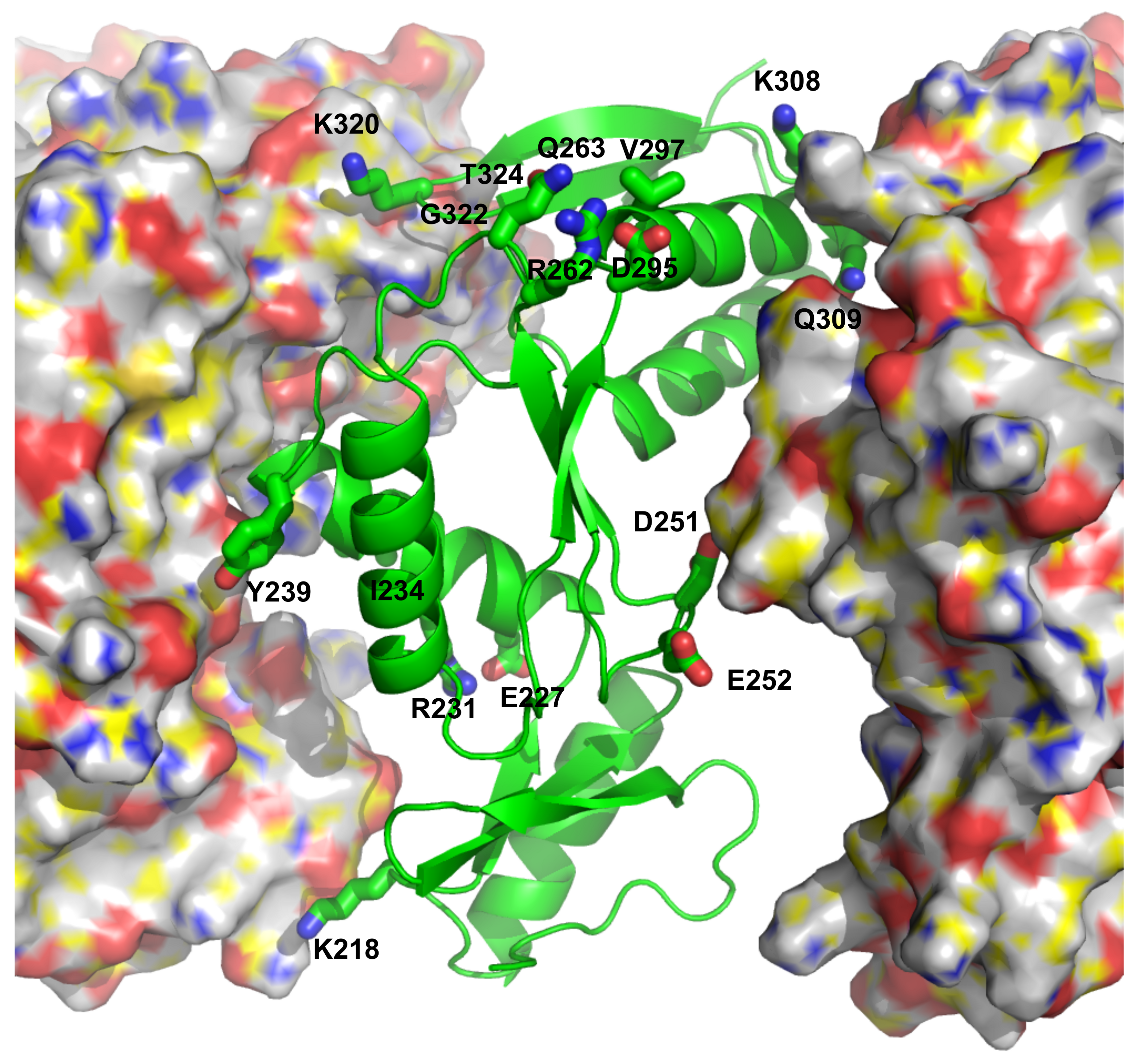

Supplement: Figure S3 — Mutants generated for the PrgH periplasmic domain. The location of all the mutated residues that were tested for secretion assay (K218A, K218L, E227L, D251A, D251L, I234A, I234L, Y239A, R231Y, I234R, Y238F, R262A, R262L, Q263A, Q263L, D295A, D295L, V297R, K308L, Q309Y, K320L, G322Y, T324H, K218A/D251A, K218A/Y239A, K218L/Y239A, K218L/Y239F, Y239A/E252A) is indicated on a PrgH molecule shown in cartoon representation, with the adjacent molecules in the ring model shown in surface representation. Only the G322Y mutant abrogated secretion (see Figure 4B) (TIF) [file ppat.1003307.s003.tif]

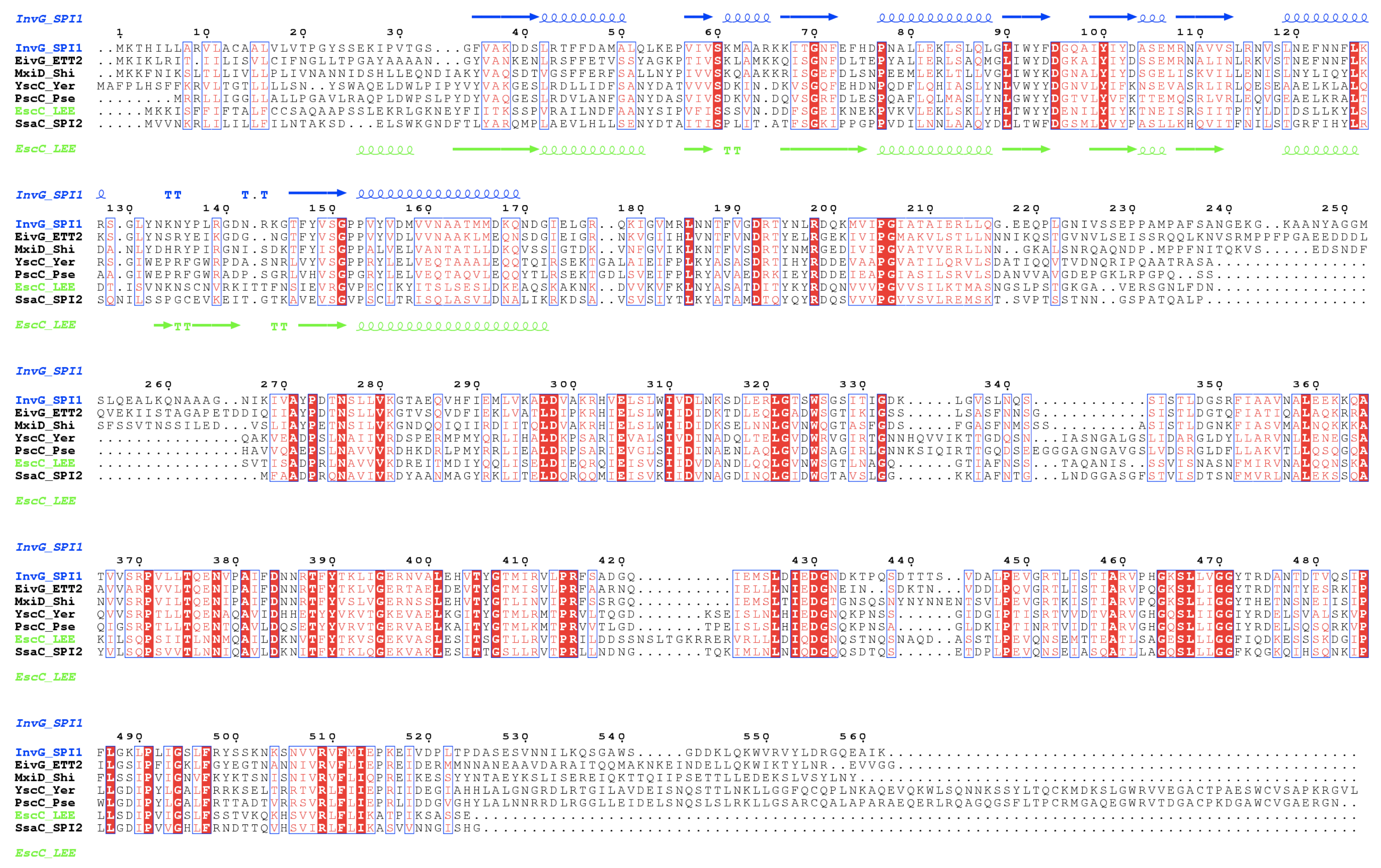

Supplement: Figure S4 — Multiple alignment of the sequences from the secretins of several T3SSs. InvG_SPI1: S. Typhimurium SPI-1 secretin InvG; EivG_ETT2: Enterohaemorrhagic E. coli ETT2 secretin EivG; MxiD_Shi: Shigella secretin MxiD; YscC_Yer: Yersinia pestis secretin YscC; PscC_Pse: Pseudomonas aeruginosa secretin PscC; EscC_LEE: Enteropathogenic E. coli LEE secretin EscC; SsaC_SPI2: S. Typhimurium SPI-2 secretin SsaC. Strictly conserved residues are in a red box, similar residues are in red characters. The secondary structures for the periplasmic domains of InvG and EscC are in blue (top) and green (bottom) respectively. (TIF) [file ppat.1003307.s004.tif]

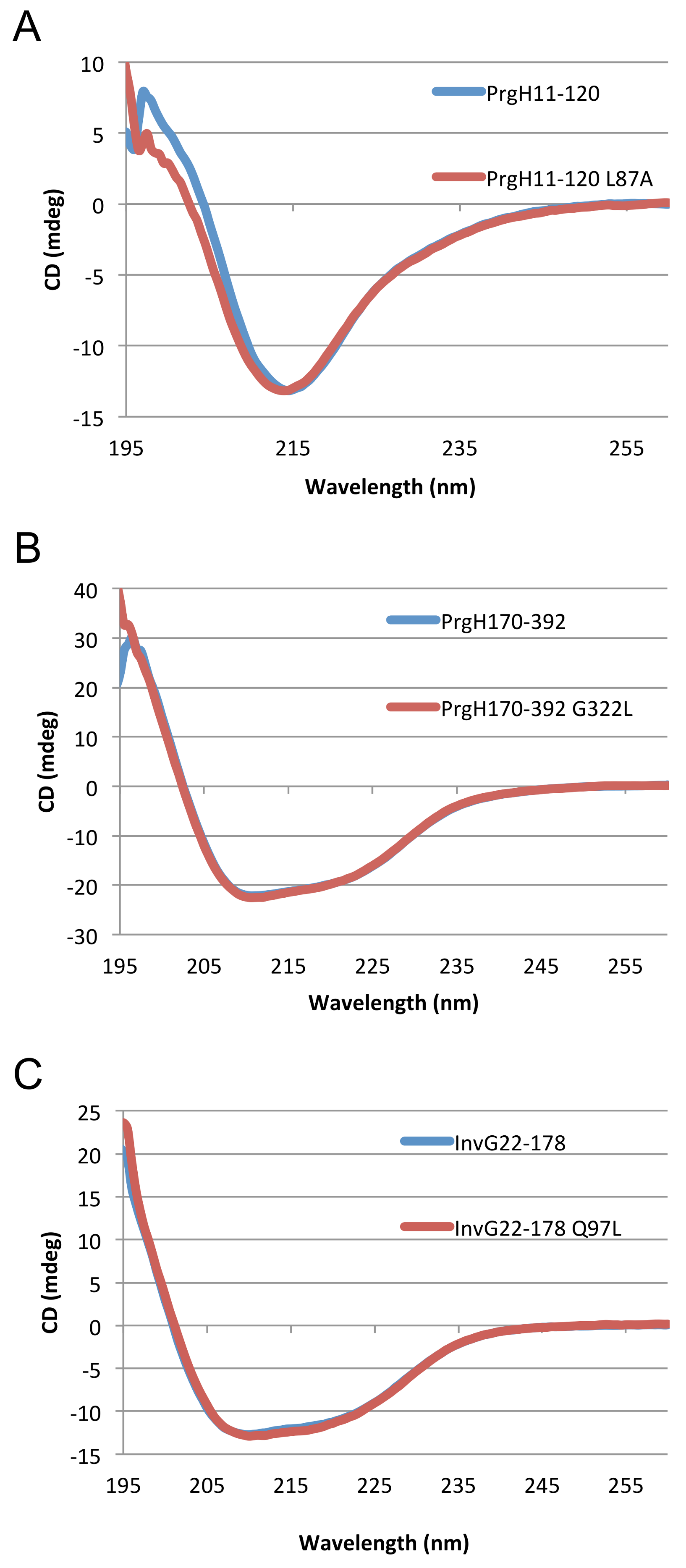

Supplement: Figure S5 — Circular dichroism analysis of InvG22–178, PrgH170–392 and PrgH11–120. Circular dichroism (CD) spectra of the crystallized domains of InvG and PrgH demonstrate the designed interface destabilizing mutants PrgH11–120 L87A (A), PrgH170–392 G322L (B) and InvG22–178 Q97L (C) have minimal effect on the overall secondary structure. CD is reported in mdeg and overlaid spectra have been corrected for protein concentration. (TIF) [file ppat.1003307.s005.tif]

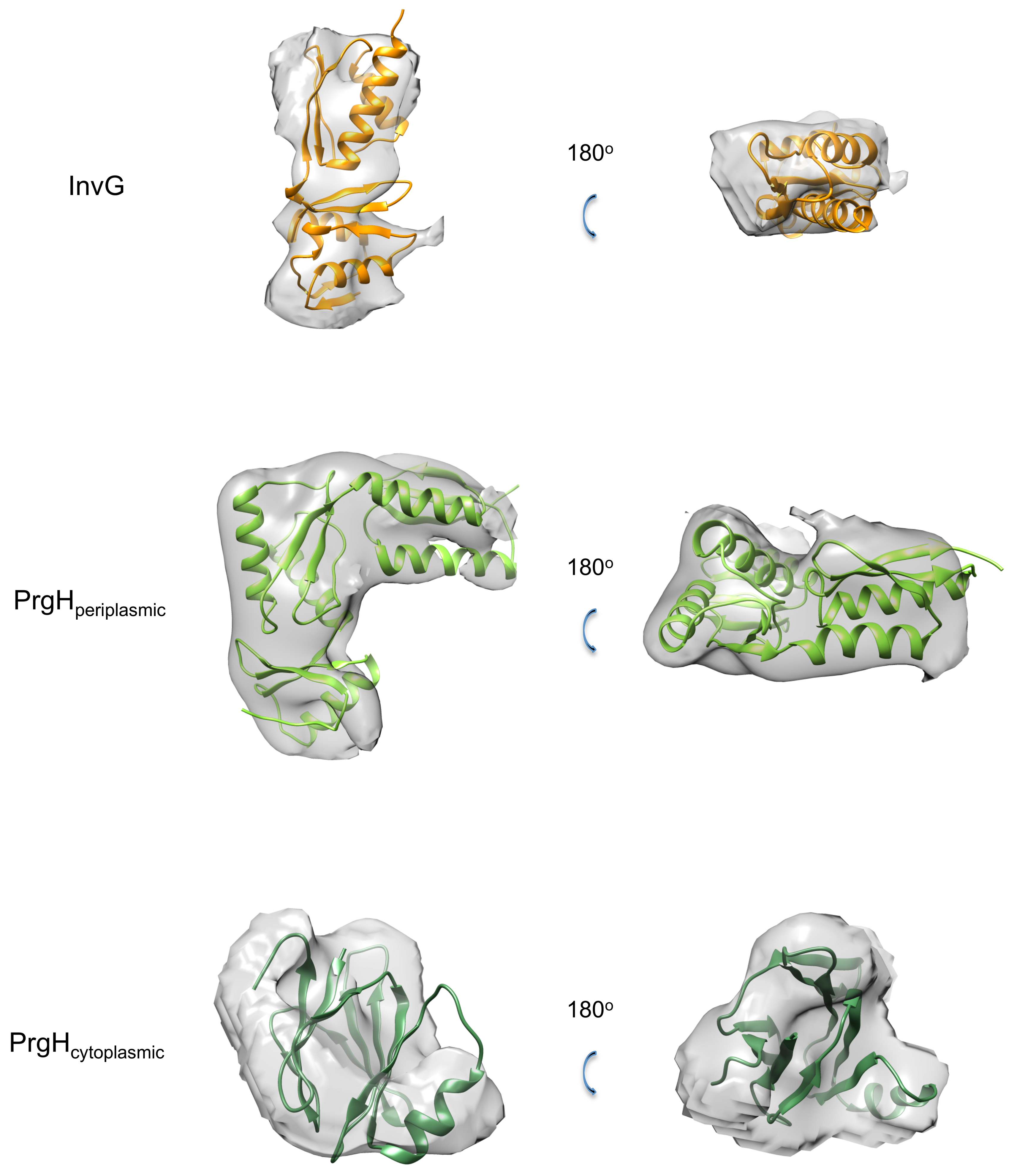

Supplement: Figure S6 — Fit of the ring models into the EM map. Close view of a monomer from the InvG (top), PrgHperiplasmic (middle) and PrgHcytoplasmic (bottom) ring models, into the EM map density (EMD-1875). Side views (left) and top views (right) are shown, and a lower contour level (0.075 for InvG, 0.08 for PegHperiplasmic and 0.045 for PrgHcytoplasmic) compared to figure 3B is used for the EM density, for closer illustration of the fit quality. (TIF) [file ppat.1003307.s006.tif]

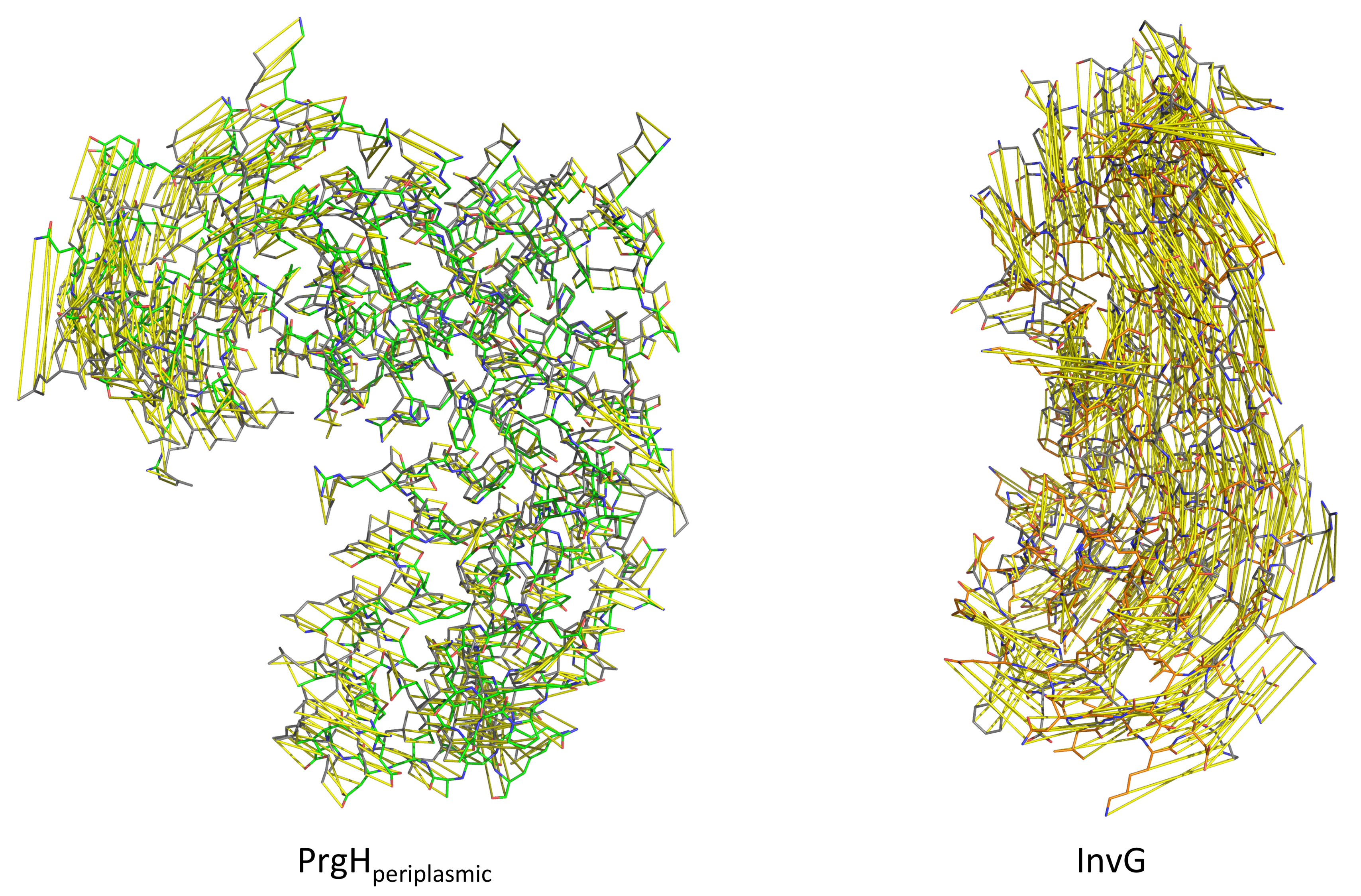

Supplement: Figure S7 — Comparison with previous models. The RMSD between monomers in our PrgHperiplasmic and InvG models (green and orange respectively) and the models reported by Schraidt and Marlovits (grey) were calculated using PyMol. The difference in atom position is shown in yellow bars, illustrating the rotation in the PrgH model and the dramatic difference in the InvG model. (TIF) [file ppat.1003307.s007.tif]

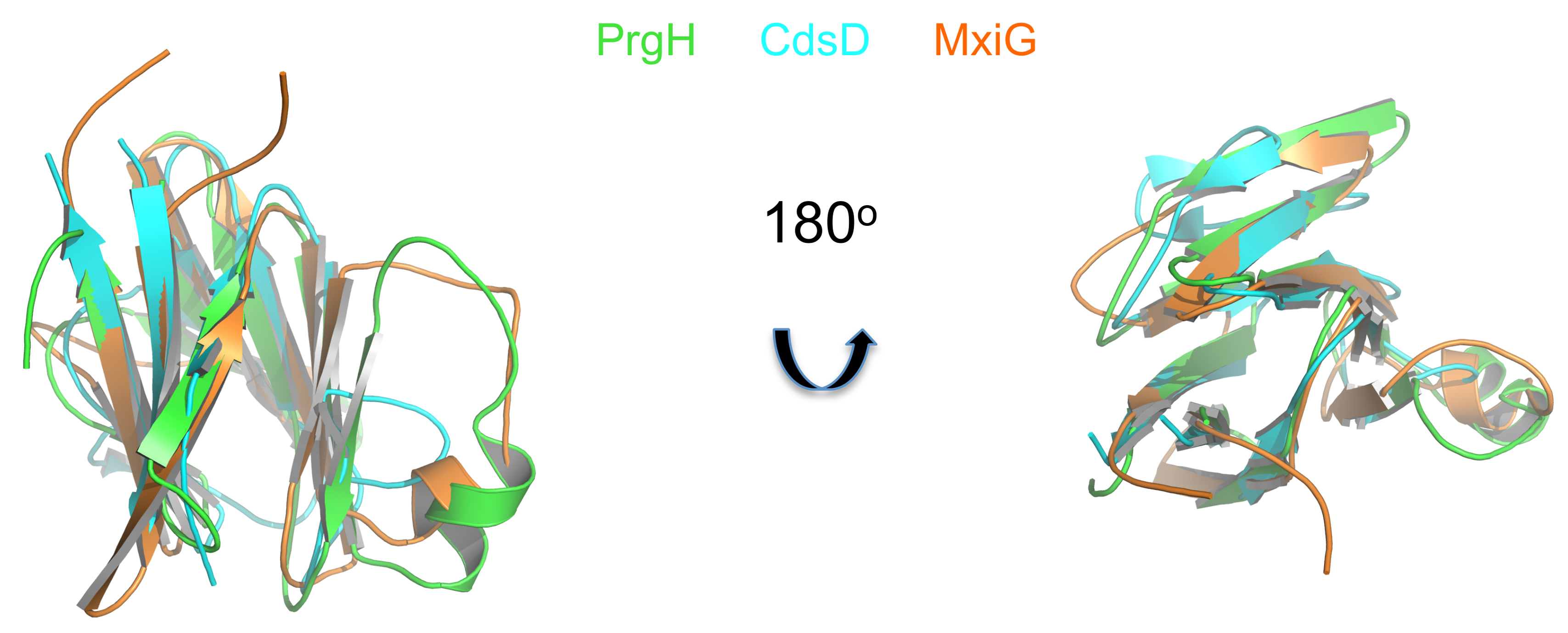

Supplement: Figure S8 — Comparison of the C-terminal domain or PrgH with its orthologues. Ribbon representation of PrgH11–120 (green), overlaid with the structures of its orthologues MxiG (pdb:2XXS, orange) from Shigella, and CT664/CdsD (pdb: 3GQS, blue) from Chlamydia, viewed from the side of the beta-sandwich (left) and from the top (right). The common FHA fold is illustrated, with the variable single helix clearly apparent. (TIF) [file ppat.1003307.s008.tif]

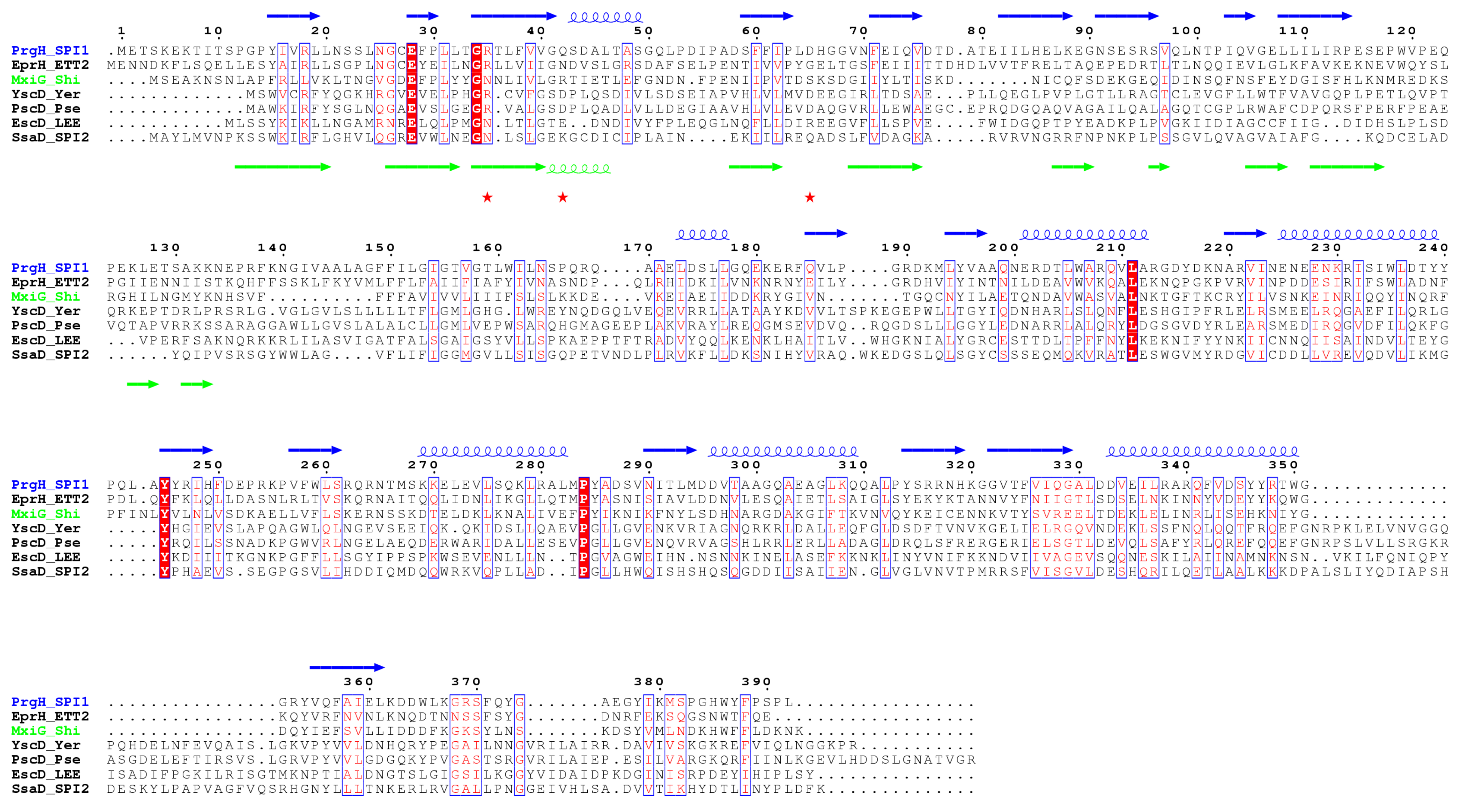

Supplement: Figure S9 — Multiple alignment of the sequences from PrgH orthologues in several T3SSs. PrgH_SPI1: S. Typhimurium SPI-1 protein PrgH; EprH_ETT2: Enterohaemorrhagic E. coli ETT2 protein EprH; MxiG_Shi: Shigella protein MxiG; YscD_Yer: Yersinia pestis protein YscD; PscD_Pse: Pseudomonas aeruginosa protein PscD; EscD_LEE: Enteropathogenic E.coli LEE protein EscD; SsaD_SPI2: S. Typhimurium SPI-2 protein SsaD. Strictly conserved residues are in a red box, similar residues are in red characters. The secondary structure of the cytoplasmic and periplasmic domains of PrgH is shown at the top (blue), and of the periplasmic domain of MxiG at the bottom (green). Red stars indicate proposed phThr-binding residues. (TIF) [file ppat.1003307.s009.tif]

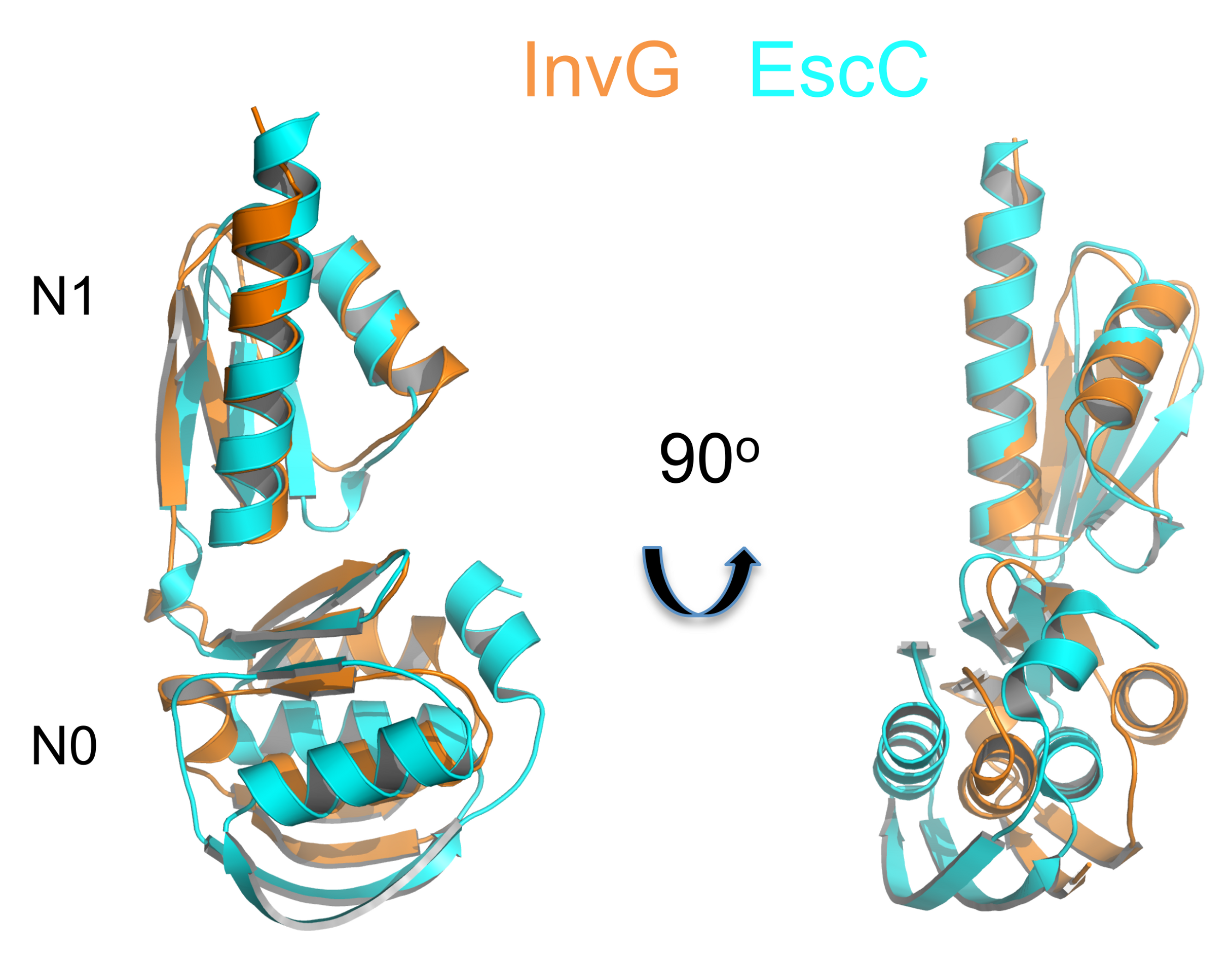

Supplement: Figure S11 — Structural differences between InvG and EscC. Ribbon representation of the structure of InvG22–178 (orange), overlaid onto the structure of EscC21–174 (cyan). The overlay was based on the N1 domains, illustrating the shift in relative orientation for the N0 domain, viewed from the side (left) or front (right). (TIF) [file ppat.1003307.s011.tif]

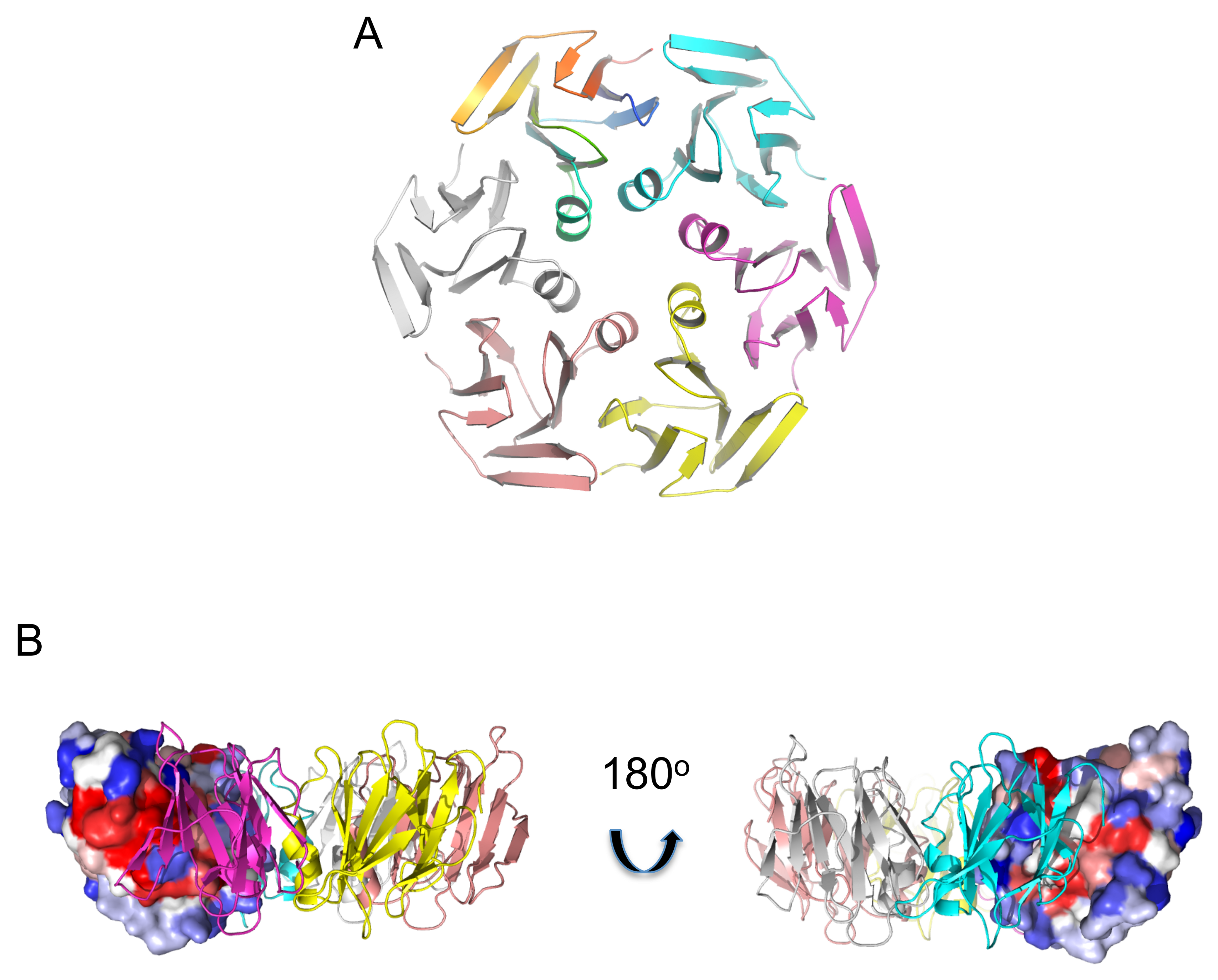

Supplement: Figure S12 — PrgH11–120 crystallizes as a hexamer. Ribbon representation of the PrgH11–120 crystal asymmetric unit. (A) The six molecules form a ring, with the helix located in the lumen. (B) Residue conservation is indicated on the surface representation, with the most conserved residues shown in red and the least conserved residues in blue. Conserved residues are primarily located at the interface between molecules, suggesting that this interface is biologically relevant. (TIF) [file ppat.1003307.s012.tif]

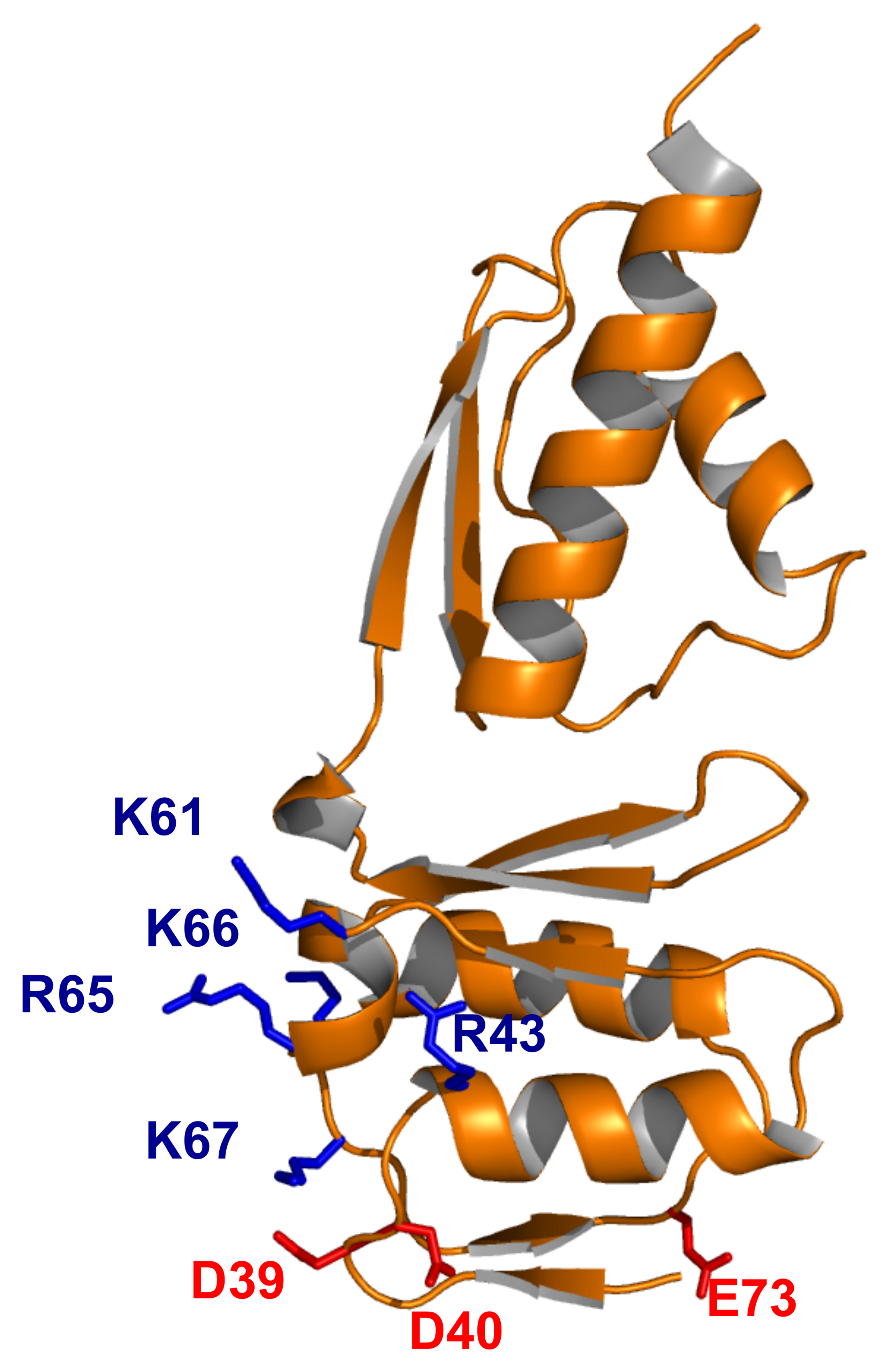

Supplement: Figure S13 — Molecular basis for the charge repartition in the InvG ring model. Ribbon representation of the InvG22–178 monomer, with the residues forming the basic lumen of the InvG ring shown in blue, and those forming the acidic patch at the bottom of the InvG ring shown in red. (TIF) [file ppat.1003307.s013.tif]
