# Supplementary material for: A Refined Model of the Prototypical Salmonella SPI-1 T3SS Basal Body Reveals the Molecular Basis for Its Assembly
Source: PLoS Pathog. 2013 Apr 25;9(4):e1003307. doi: 10.1371/journal.ppat.1003307 (PMC3635987; doi:10.1371/journal.ppat.1003307)
Supplement: Appendix S1 — Detailed protocol for the two-step modelling procedure. (A) Schematic representation of the procedure employed. (B) Command lines used in Rosetta for the modelling procedure. (DOC) [file ppat.1003307.s014.doc]

**APPENDIX S1**

**A. Overview of the two-step approach.**

**
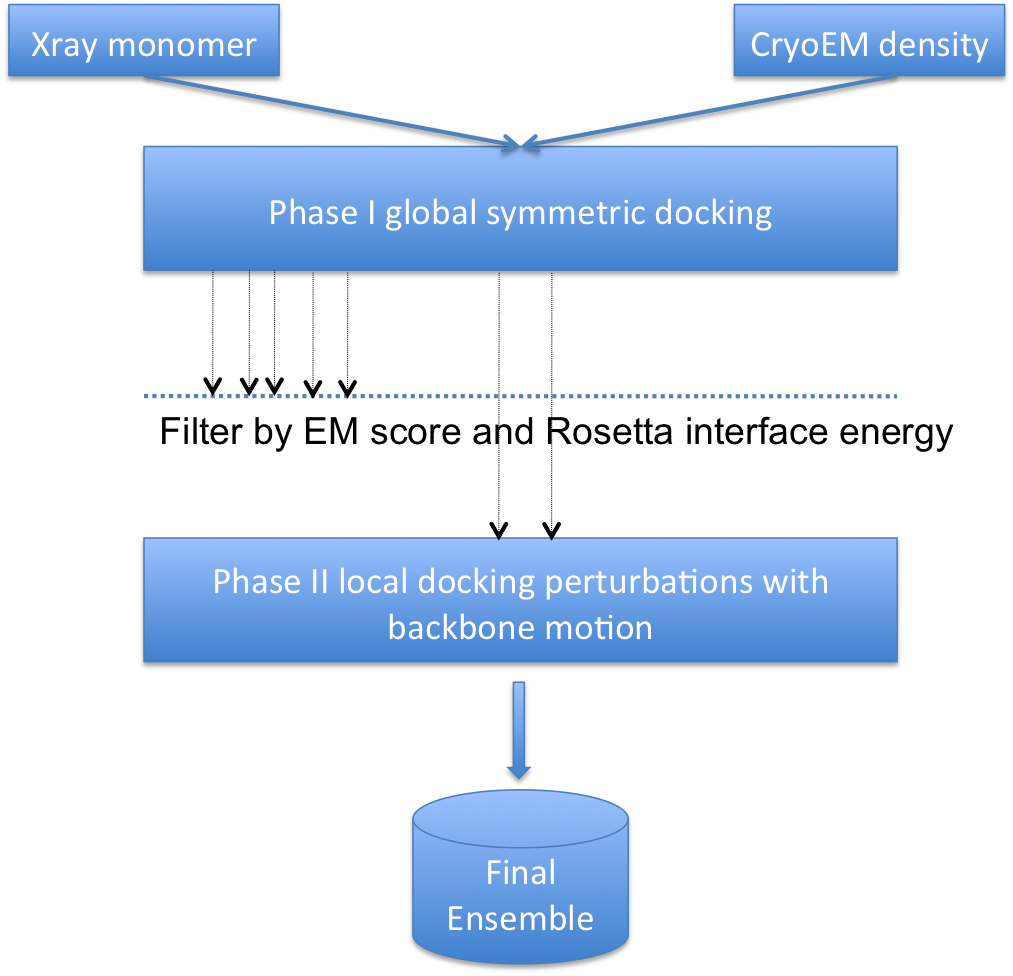
**

**B. Command lines for execution of the two-step docking protocol.**

These command lines are compatible with ROSETTA 3 SVN version 51540M <https://svn.rosettacommons.org/source/trunk/rosetta/rosetta_source>.

**Step 1: Global symmetric docking**

minirosetta.static.linuxgccrelease \

-run:protocol symdock \

-database $rosettadir/rosetta_database \

-in:file:s monomer_input.pdb \

-symmetry:symmetry_definition C15_phaseI.symm \

-symmetry:initialize_rigid_body_dofs \

-edensity:mapfile invg.mrc \

-edensity:mapreso 10.0 \

-edensity:grid_spacing 5.0 \

-edensity:score_symm_complex true \

-packing:ex1 \

-packing:ex2aro \

-use_input_sc \

-ignore_unrecognized_res \

-out:nstruct 1000 \

-out:file:silent invg.phaseI.silent \

-out:file:silent_struct_type binary \

-out:file:fullatom \

-residues:patch_selectors CENTROID_HA \

-use_incorrect_hbond_deriv false \

-docking:low_patch patch_phaseI \

-docking:high_patch patch_phaseI \

-docking:high_min_patch patch_high_min_phaseI \

-docking:pack_patch patch_phaseI \

-docking:dock_lowres_filter 15.0 20.0 1500.0 \

**Step 2: Local symmetric perturbation starting from selected monomer pdbs from step 1 based on their Rosetta score and EM score (phaseI.pdb)**

minirosetta.static.linuxgccrelease \

-run:protocol symdock \

-database $rosettadir/rosetta_database \

-in:file:s phaseI_INPUT.pdb \

-symmetry:symmetry_definition C15_phaseII.symm \

-symmetry:perturb_rigid_body_dofs 3 5 \

-edensity:mapfile invg.mrc \

-edensity:mapreso 10.0 \

-edensity:grid_spacing 5.0 \

-edensity:score_symm_complex true \

-packing:ex1 \

-packing:ex2aro \

-use_input_sc \

-ignore_unrecognized_res \

-out:nstruct 100 \

-out:file:silent invg.phaseII.silent \

-out:file:silent_struct_type binary \

-out:file:fullatom \

-residues:patch_selectors CENTROID_HA \

-use_incorrect_hbond_deriv false \

-docking:low_patch patch_phaseII \

-docking:high_patch patch_phaseII \

-docking:high_min_patch patch_high_min_phaseII \

-docking:pack_patch patch_phaseII \

-docking:dock_lowres_filter 15.0 20.0 1500.0 \

-docking:kick_relax \

-score:weights score12_full \

-score:patch patch_phaseII \

***Patch files***

Patch files are used to set weights of the score function. Three different patch files are used to control sampling (low resolution, all atom) and selection into the pool of structures.

==> patch_phaseI<==

elec_dens_whole_structure_ca = 0.1

==> patch_high_min_phaseI<==

elec_dens_whole_structure_ca = 0.1

fa_rep *= 4.22

==> patch_phaseII <==

elec_dens_whole_structure_ca = 0.01

==> patch_high_min_phaseII <==

elec_dens_whole_structure_ca = 0.01

fa_rep *= 4.22

***Generation of Symmetry Definition Files (example shown for the InvG 15mer ring)***

==> C15.phaseI.symm <==

$rosettadir/rosetta_source/src/apps/public/symmetry/make_symmdef_file_denovo.py -nsub 15\

–symm_type cn > C15.phaseI.symm

The command will generate a generic SDF for C15 symmetry

==> C15.phaseII.symm <==

perl $rosettadir/rosetta_source/src/apps/public/symmetry/make_symmdef_file.pl -a A -i B -r 12.0 \

–p phaseI.pdb > C15.phaseII.symm

This command will also generate the input monomer model phaseI_INPUT.pdb to be used for input in phase II docking (see commands in section 1).

This command will extract the symmetry transformations from an input pdb file to be used in further Rosetta refinement in Phase II. Repeating the command for different pdb input files will allow for phase II evaluation of candidate ring arrangements from phase I.
